# A tumor microenvironment responsive nanosystem for chemodynamic/chemical synergistic theranostics of colorectal cancer

**DOI:** 10.7150/thno.61651

**Published:** 2021-08-18

**Authors:** Liying Wang, Jingya Xia, Hongjie Fan, Min Hou, Huiyang Wang, Xiaoyan Wang, Ke Zhang, Liping Cao, Xiangrui Liu, Jun Ling, Hong Yu, Xia Wu, Jihong Sun

**Affiliations:** 1 Department of Radiology, Sir Run Run Shaw Hospital, Zhejiang University School of Medicine, Hangzhou, 310016, China.; 2 Department of General Surgery, Sir Run Run Shaw Hospital, Zhejiang University School of Medicine, Hangzhou, 310016, China.; 3 MOE Key Laboratory of Macromolecular Synthesis and Functionalization, Department of Polymer Science and Engineering, Zhejiang University, Hangzhou, 310027, China.; 4 Cancer Center, Zhejiang University, Hangzhou, Zhejiang, 310058, China.

**Keywords:** Chemodynamic therapy, Nanotheranostics, Microenvironment-responsive, Magnetic resonance imaging, Synergistic therapy

## Abstract

**Rationale:** The synergism of new modalities alongside chemodynamic therapy into common chemotherapy has shown promising potential in clinical applications. This paper reports a tumor microenvironment-responsive nanosystem for chemodynamic/chemical synergistic therapy and magnetic resonance imaging (MRI).

**Methods:** The biodegradable nanosystem is synthesized using a surface-modified chain transfer agent for surface-initiated living radical polymerization of the chemotherapeutic drug.

**Results:** In this nanosystem, named CAMNSN@PSN38, the cycling time and solubility of the chemotherapeutic drug are improved. The nanoparticles delivered to tumor tissues gradually release the chemotherapeutic drug and Mn^2+^ through glutathione (GSH)-triggered biodegradation in the tumor microenvironment. SN38, the released chemotherapeutic drug, not only shows excellent chemical therapy effects but also improves the generation of H_2_O_2_. Furthermore, with the Fenton-like agent Mn^2+^, the generation of reactive oxygen species (ROS) is improved markedly. Finally, CAMNSN@PSN38 shows excellent inhibition of tumor growth in three colorectal cancer tumor models, with an improved accumulation of ROS and controlled release of SN38.

**Conclusions:** The CAMNSN@PSN38-mediated chemodynamic/chemical synergistic therapy provides a promising paradigm for the treatment and MRI-guided therapy of colorectal cancer.

## Introduction

Colorectal cancer (CRC) is one of the most commonly diagnosed cancers worldwide, moreover, among cancer it has the second-highest mortality. In 2018, approximately 1.096 million cases of CRC were diagnosed, with 551,269 deaths 1. The early diagnosis and effective anticancer strategy for CRC remain challenging. Common clinical strategies such as chemotherapy, radiotherapy, and surgical resection have been widely applied in the treatment of CRC 2. However, the clinical effects of chemotherapy and radiotherapy are always accompanied by unsatisfactory drug delivery, drug resistance, poor anti-tumor performance, and severe side effects caused by systemic toxicity 3, 4, which can even lead to tumor metastases 5. Surgical procedures, the most used treatment for CRC, suffer from metastases 6, 7. Accordingly, novel and effective anti-cancer strategies for the theranostics of CRC are urgently needed.

Over the past several decades, numerous novel anticancer therapies have been designed based on the intricacy of the tumor microenvironment (TME) 8-11. The TME is known to present several characteristics such as overproduction of hydrogen peroxide (H_2_O_2_, ≈ 50-100 × 10^-6^ M) and glutathione (GSH, ≈10 × 10^-3^ M) 12. As a reactive oxygen species (ROS)-mediated therapy, chemodynamic therapy (CDT) 13-15 has been developed, takes advantage of Fenton or Fenton-like reactions *in situ,* uses transition metal ions source to generate cytotoxic hydroxyl radicals (•OH) from the action of H_2_O_2_ 16. Several innovative ideas 17, including utilizing intracellular ions as catalyst source 18 and using liposomal lipid membrane to protect generated •OH from eliminating by GSH 19, were put forward to improve the chemodynamic therapy. Chemotherapy, which is one of the most viable treatment options in CRC therapy, utilizes highly cytotoxic drugs to kill tumor cells. However, severe side effects and poor drug delivery are damaging to clinical applications and outcomes 20. Recently, several studies have used nanomedicine to combine chemotherapy with chemodynamic therapy to inhibit tumor growth 21 and suppress metastases 22, 23. Nevertheless, the low amount of H_2_O_2_ and the relatively high concentration of GSH limits both the accumulation of ROS and the efficiency of the novel combination therapy 16, 24.

The problem of relatively low levels of H_2_O_2_ in tumor tissues can be solved through common strategies which include directly loading H_2_O_2_ or encapsulating additional H_2_O_2_ - generating cargos such as glucose oxidase 22 and calcium peroxide 25. However, H_2_O_2_ leakage was observed in nanocarriers that directly encapsulated exogenous H_2_O_2_, which was proven to cause damage to normal tissues 23. Furthermore, the delivery of glucose oxidase and calcium peroxide was complex and the reaction of generating H_2_O_2_ was uncontrollable in the delivery processes 16. Therefore, novel strategies are urgently needed to solve the challenges associated with chemotherapy/chemodynamic synergistic therapy.

ROS is relatively abundant in tumor tissues, with concentrations as high as 100 μM, while in normal tissue this was 20 nM 26. However, reactive species such as reductive glutathione maintain the balance of oxidative stress in cells 27. The current reported strategies are based on eliminating the influence of GSH, including delivering GSH scavengers 28 and inhibiting the synthesis of GSH with nanocarriers 29. The additional cargos in the nanocarriers increase the complexity of the delivery system and the leaking of scavengers harm normal tissue. Recently, a biodegradable MnSiO_3_ nanosystem was reported, which could react with GSH and release Mn^2+^ for magnetic resonance imaging (MRI) 30. Therefore, depletion of GSH with the nanocarrier, but not the addition of GSH scavengers, is a promising strategy in chemotherapy/chemodynamic synergistic therapy.

The active metabolite of irinotecan, 7-ethyl-10-hydroxycamptothecin (SN38), is the first-line drug in the treatment of CRC with poor solubility in pharmaceutically approved solvents 31. Hence, the delivery of SN38 has been difficult in CRC therapy. In this study, a biodegradable nanosystem of MnSiO_3_ for chemodynamic/chemical synergistic therapy and MRI was reported. The biodegradable MnSiO_3_ nanosystem was synthesized, and the surface was modified with a chain transfer agent *via* the reversible addition-fragmentation chain transfer (RAFT) reaction. Finally, the surface-initiated living radical polymerization of MMESSN38 (monomer of SN38) and oligo(ethylene glycol) methacrylate (OEGMA) was conducted to obtain the product CAMNSN@PSN38. In this nanosystem, the cycling time and solubility of SN38 were improved. The nanoparticles delivered to the tumor tissue could be gradually biodegraded with GSH over time, during which SN38 and Mn^2+^ were gradually released. Interestingly, the released SN38 showed an excellent chemical therapy effect and improved the accumulation of H_2_O_2_. The interaction between CAMNSN@PSN38 and GSH not only consumed GSH in tumor tissue but also led to the release of MR and Fenton-like dual-functional agent Mn^2+^ for chemodynamic therapy and MRI-guided therapy. CAMNSN@PSN38 showed excellent inhibition of CRC tumor growth as well as assisting MR-guided imaging ability *in vivo* with the accumulation of ROS.

## Materials and methods

### Instruments

TEM images and energy-dispersive spectra were obtained using an HT-7700 electron microscope (Hitachi Ltd) equipped with an X-MAXn65T CCD camera (Oxford Instruments). XRD was performed using a Bruker D8 ADVANCE instrument. XPS was conducted using a Thermo Scientific ESCALAB 250Xi. The absorption and fluorescence spectra were detected using a Spectramax M5 (Molecular Devices). Dynamic light scattering was performed using a Malvern Zetasizer Nano-ZS to measure the size distributions of the obtained nanoparticles. The acquisition of size data was performed according to the manufacturer's protocols. The nitrogen adsorption-desorption isotherm curves were measured by a pore size and sorption analyzer (AUTOSORB-IQ2-MP, Quantachrome, USA). The systems comprised a laser operating at 633 nm and a multiple tau digital correlator electronics system.

### Materials

Tetraethyl orthosilicate (TEOS), cetyltrimethylammonium bromide (CTAB), CH_3_COONa, MnCl_2_, urea, triethanolamine, 3-aminopropyltriethoxysilane (APTES), FITC, N-hydroxysuccinimide, N-ethyl-N'-(3-dimethylaminopropyl) carbodiimide (EDC), 4-Dimethylaminopyridine (DMAP), and 2,2'-azobis (2-methylpropionitrile) (AIBN) were purchased from Macklin Chemical Reagent Co. Ltd. OEGMA (M.W.=500 Da) was purchased from Sigma Chemical Reagent Co. Ltd. 4',6-Diamidino-2-phenylindole (DAPI) was purchased from Sigma-Aldrich. Co. (USA). Cell counting kit-8 (CCK-8) was purchased from Dojindo (Japan). All chemical reagents were pure in grade and were used directly without further purification. MMESSB38 was synthesized according to previous research in our laboratory 32.

### Synthesis of MSN

The MSN (mesoporous silica nanoparticle) was synthesized according to a previous study, but with modifications 30. Briefly, urea (15 g) and CTAB (0.814 g) were mixed and added to deionized water (50 mL) and stirred at 80 °C for 0.5 h to obtain a clear solution. Then, TEOS solution (150 mL) was added slowly. The mixture was continuously stirred for an additional 24 h at 80 °C. Crude MSNs were obtained by centrifuging the mixture at 11000 rpm for 10 min and dried overnight. In order to remove CTAB, the crude products were dispersed in acetone and refluxed at 70 °C for 12 h. The product was obtained through centrifugation at 16000 rpm for 30 min and dried overnight (white powder, 1.2 g).

### Synthesis of MNSN

MNSN (manganous silicate nanoparticle) was synthesized according to a previously reported method 25. The MSNs (0.8 g) were well dispersed with ultrasound in deionized water (40 mL), following which MnCl_2_ (2.4 g) and CH3COONa (0.4 g) were added and the pH was adjusted to 7.5 under stirring with the addition of triethanolamine. The obtained mixture was then added to a 100 mL Teflon-lined stainless-steel autoclave and placed in an oven at 110 °C for 48 h. MNSN was obtained by centrifuging at 4500 rpm for 15 min. (brown powder, ≈0.6 g, yield: 75%).

### Synthesis of AMNSN

AMNSN (amino-modified MNSN) was synthesized according to previous research on manufacturing amino-modified MSNs, with modification 33. MNSN (0.8 g) was well dispersed in toluene (80 mL) under ultrasonication, following which APTMS (0.8 mL) was rapidly added. The resulting reaction solution was stirred under condensation at 60 °C for 9 h. In order to remove the unreacted modifier (APTMS), the obtained mixture was washed with ethanol three times and dried at 60 °C overnight. The obtained deep brown powder was named AMNSN (deep brown powder, ≈0.56 g, yield: 70%).

### Synthesis of CAMNSN

RAFT** (**reversible addition-fragmentation chain transfer) chain transfer agent-modified MNSN (CAMNSN) was synthesized according to previous research on manufacturing RAFT-MSN with modification 34. CAMNSN (0.32 g) was reacted with 4-cyanopentanoicacid dithiobenzoate (279 mg) in the presence of EDC (95.7 mg) and DMAP (17.51 mg) in anhydrous dichloromethane (100 mL) at room temperature for 48 h. After the reaction, CAMNSN was obtained by centrifuging at 4500 rpm for 15 min and then washing with DCM and methanol three times to remove the unreacted RAFT agents. The resulting brown material was dried under vacuum overnight (brown powder, ≈0.24 g, yield: 75%).

### Synthesis of CAMNSN@PSN38

CAMNSN (50 mg), AIBN (1 mg), OEGMA (M.W.=500 Da, 0.75 g), and MMESSB38 (0.25 g) were mixed in a Schlenk tube with dioxane (2 mL) and anhydrous dimethyl sulfoxide (DMSO, 2 mL), followed by sonication for 5 min to obtain a homogenous suspension. The tube was subjected to three cycles of a freeze-pump-thaw process to remove oxygen and then placed in an oil bath at 70 °C for 24 h. The polymerization of OEGMA and MMESSN38 was terminated by exposing the tube to the atmosphere and placing the tube in ethanol to return to room temperature. Subsequently, the obtained mixture was diluted with DMSO, and then dialyzed with DMSO and deionized water five times. The resulting CAMNSN@PSN38 was obtained by lyophilization (deep brown solid, 720 mg, yield: 69%).

### FITC Labeled CAMNSN@PSN38

FITC (20 mg) and N-hydroxysuccinimide (5 mg) were mixed in DMSO (2 mL) and incubated at 37 °C overnight in an argon atmosphere. Then, CAMNSN@PSN38 (50 mg) in DMSO (2 mL) was injected under an argon atmosphere and stirred for another 48 h. Subsequently, the obtained solution was dialyzed with DMSO (3 × 250 mL) and water (3×1 L). After lyophilization, FITC-labeled CAMNSN@PSN38 was obtained.

### Release Behavior of CAMNSN@PSN38

CAMNSN@PSN38 (0.5 mg) was well dispersed in phosphate-buffered saline (PBS, 1 mL, pH = 7.4) plus Tween 80 (0.5%) with or without GSH (1 mM or 10 mM). Then, the obtained solutions were placed in a dialysis bag, and the bag was immersed in PBS solution (100 mL) with or without GSH (1 mM or 10 mM) in a tube. The tube was shaken at 200 rpm at 37 °C, and supernatant (1 mL) was removed at different time intervals for measurement of Mn and SN38. The release behavior of Mn in mildly acidic solution was measured with a similar method in PBS solution (pH = 5.5). The amount of Mn released from the CAMNSN@PSN38 was determined by using inductively coupled plasma optical emission spectrometry and calculated using this equation: Released Mn (%) = concentration (Mn) × volume / amount (Mn) × 100%. The amount of SN38 released from the CAMNSN@PSN38 was determined by HPLC and calculated using this equation: Released SN38 (%) = concentration (SN38) × volume / amount (SN38) × 100%.

### *In vitro* MRI Imaging Properties

In all, the well-dispersed solution of CAMNSN@PSN38 (100 μL, concentration of Mn = 5 mM) was added to PBS solution (900 μL, pH= 7.4) with or without GSH (10 mM) and then shaken at 37 °C for 2 h. Then, MR images and the T1 relaxation time of MnCl_2_, diluted supernatant of CAMNSN@PSN38 treated with GSH or not (Mn concentration: 0.16, 0.32, 0.48, 0.64, and 0.80 mM), were measured using a 3.0 T MRI system with inversion recovery (IR) pulse sequence.

### *In vivo* MRI Imaging

A CT26 cell line transplant subcutaneous mouse model was produced and used. When the tumor volume reached approximately 200 mm^3^, *in vivo* MR imaging experiments were conducted. CAMNSN@PSN38 (e.q. [SN38]: 10 mg kg^-1^) was injected intratumorally or *via* the tail vein and T1-weighted MR images were recorded on a 3.0 T MRI system (Signa HDxt, GE Medical Systems, Milwaukee, WI, USA) with a specialized mouse coil at different time points. The T_1_-weighted spin-echo sequence parameters were set as follows: T_R_/T_E_=500/10 ms, matrix= 288× 256, slice thickness = 1.5 mm, spacing = 0 mm, and FOV= 6 × 4.5 cm^2^.

### ESR measurement

DMPO (5,5-Dimethyl-1-pyrroline-N-oxide, 50 mM) was added into PBS buffer solution (pH = 5.5) containing CAMNSN@PSN38 (e.q. [Mn] = 25 ppm), followed by treating with or without H_2_O_2_ (50 μM). After 10 s of reaction, the obtained solution was transferred to a quartz tube and the ESR signal was obtained on a spectrometer.

### Cell Culture

All colorectal cancer cell lines were purchased from ATCC and cultured in 75 cm^2^ culture flasks in a cell incubator at 37 °C and 5% CO_2_. According to the manufacturer's s specifications, CT26-luc was cultured in Roswell Park Memorial Institute (RPMI) 1640 (Gibco), supplemented with 10% fetal bovine serum (FBS). HT29 cells were cultured in McCoy's 5a Medium (Gibco) supplemented with 10% FBS and Pen-Strep. The SW620 cell line was cultured in Dulbecco's modified Eagle's medium (DMEM, Gibco) supplemented with 10% FBS and Pen-Strep. The culture medium was changed every three days. The cells were collected, counted via CountStar, and assessed for viability (> 95% viable) for potential experimental use.

### Cytotoxicity Assay *in vitro*

The cytotoxicity of MNSN and CAMNSN@PSN38 against tumor cell lines was quantified by the Cell Counting Kit-8(CCK-8) assay. Cells were seeded at a density of 5 × 10^3^ per well in 96-well plates and incubated overnight, followed by the addition of MNSN and CAMNSN@PSN38 at different concentrations. The cells were then cultured for a further 48 h. Subsequently, the drug-containing culture medium was replaced with a complete medium (100 μL) containing CCK-8 solution (10 μL). Three hours later, the absorbance at 450 nm was recorded using a microplate reader and the cell viability was calculated according to the manufacturer's instructions. All experiments were performed in triplicates.

### Flow Cytometry Study

Briefly, CT26 cells (2×10^5^ cells per well) were plated in 12-well plates and incubated overnight. Then, FITC-labeled CAMNSN@PSN38 (at a final e.q. [SN38] = 5 µg mL^-1^) was added and incubated for 2, 4, and 6 h. Then, the cells were obtained and washed with PBS three times before observation by a CytoFLEX LX flow cytometer (Beckman Coulter, Brea, CA, USA). The data were analyzed using the FlowJo software.

### Subcellular Distribution

To observe the subcellular uptake of CAMNSN@PSN38, CT26 cells were seeded at a density of 1 × 10^5^ in glass-bottom dishes in a complete culture medium (1 mL, RPMI 1640, supplemented with 10% FBS, GIBCO) overnight. The cells were then incubated with fresh medium containing FITC-labeled CAMNSN@PSN38 ([SN38] = 5 µg mL^-1^) for 2, 4, and 6 h at 37 °C. Subsequently, the cells were washed and stained with Hoechst 33342 and Lysotracker green (Meilunbio, China) for 15 min and were evaluated by confocal laser scanning microscopy (CLSM, Leica, TCS SP8, US).

### Measurement of H_2_O_2_ Generation

The H_2_O_2_ content in treated cells was analyzed with a Hydrogen Peroxide Assay Kit (Beyotime, S0038, China). CT26 cells were seeded in 25-cm^2^ culture flasks and incubated overnight. Then, SN38 solutions (0, 10, 100, and 1000 ng mL^-1^) were added to the culture medium and incubated for an additional 24 h. The cells were collected, lysed, and mixed with detection reagent, then the absorbance was measured according to the protocol of the Hydrogen Peroxide Assay Kit. Absorbance values were measured at a wavelength of 560 nm and uniformized with the control value, moreover, the cell number was counted by a cell counter (Countstar® BioTech, China).


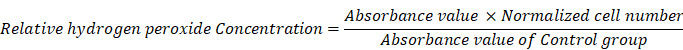


### Measurement of ROS Generation

ROS generation was measured using a microplate reader and CLSM with dichloro-dihydro-fluorescein diacetate (DCFH-DA). Upon oxidation by ROS and cleavage of the esterase, DCFH-DA turns into a highly fluorescent molecule, DCF.

### Measurement by a Microplate Reader

CT26 cells were seeded at a density of 4 × 10^3^ per well in 96-well plates and cultured overnight. Then, different distributions of CAMNSN@PSN38 or SN38 solutions (0, 1, 5, 10, 20, 50, 75, 100, and 200 µM) were added. After 24 h, the medium containing DCFH-DA (10 µM) was replaced and incubated for another 3.5 h. The absorbance values were then measured using a microplate reader (Molecular Devices) at a wavelength of 530 nm.

### Measurement by CLSM

CT26 cells were seeded at a density of 1 × 10^5^ in glass-bottom dishes in a complete culture medium (1 mL, RPMI 1640, supplemented with 10% FBS, GIBCO) overnight. The cells were then incubated with fresh medium containing CAMNSN@PSN38 (at a final e.q. [SN38] = 20 µM) for an additional 24 h at 37 °C. Images were taken using a CLSM (Leica, TCS SP8, US). ROS (fluorescence of DCF) was measured using a 488 nm laser and expressed as green fluorescence.

### Antitumor Activity

All animal experiments were conducted following protocols approved by the Zhejiang University Laboratory Animal Center following the guidelines for the care and use of laboratory animals. Female BALB/c nude mice were purchased from Shanghai SLAC Laboratory Animal Co., Ltd. and maintained under standard conditions.

Human colorectal cancer HT29 cells (5 × 10^6^) or murine colorectal cancer CT26 cells (1 × 10^6^) suspended in PBS (200 µL) were subcutaneously injected into the left flank of each BALB/c mouse (6 - 8 weeks old). To develop the xenografted peritoneal metastatic colorectal cancer model, BALB/c mice were intraperitoneally (i.p.) injected with 1 × 10^6^ murine colorectal cancer CT26 cells which were stably expressing luciferase (CT26-Luc) suspended in PBS (200 µl). Tumor-bearing mice were randomized into five groups seven days after injection and treated with antitumor therapy three times by intravenous administration with PBS, MNSN, CPT-11, a physical mixture of MNSN with CPT-11, or CAMNSN@PSN38 at the SN38 dose of 10 mg kg^-1^. Tumor growth and the body weight of mice were monitored every other day. The tumor size was estimated using the following formula: length × width^2^ × 0.5. *In vivo* luciferase bioluminescence was measured via the imaging system (IVIS, PerkinElmer) on day 0, 5, 10, and 15. Five minutes after the i.p. injection of D-luciferin (150 mg kg^-1^), the mice were imaged and the tumor photometry was analyzed using Living Image 3.1.0. The CT26-luc subcutaneous tumor-bearing mice were sacrificed on day 15 of the antitumor treatment, then the major organs and tumor tissues were harvested for f H&E, TUNEL, and ROS staining analysis.

### Histochemical and Immunofluorescence Assays

For histochemical studies, tumor tissue sections (6 µm) were obtained and stained with TUNEL (Roche Co., REF 11684817910) and H&E (Beyotime Co., China) following the manufacturer's instructions. Tumor tissue ROS staining was performed using a dihydroethidium stain according to previous studies with minor modifications 35.

### HPLC Analysis

The concentrations of SN38 were measured by an Agilent Technologies 1260 Infinity II HPLC system consisting of a quaternary HPLC pump, an auto-sampler, a fluorescence detector, and a photodiode array detector. A ZORBAX Eclipse XDB-C18 column (4.6 × 250 mm, 5 µm) was used in the HPLC system. The OpenLAB CDS for the LC System was used for the acquisition and analysis of data. CAMNSN@PSN38 was disintegrated by an equal volume of NaOH solution (0.1 M) at 37 °C overnight. Then, the solution was diluted 4-fold with methanol and an equal volume of HCl (0.1 M) before analysis. A sample (80 µL) was injected into the column at 40 °C. The mobile phase consisted of a mixture of trifluoroacetic acid aqueous solution (0.1%) and methanol (40:60, v/v%) at a total flow rate of 1 mL min^-1^. SN38 was detected using a fluorescence detector (Ex/Em wavelengths: 360 nm/543 nm).

### Biosafety and Pharmacokinetics Study

Female ICR mice were divided into three groups (n=5) and injected with PBS, MNSN (5 mg kg^-1^), and CAMNSN@PSN38(SN38-eq dose at 10 mg kg^-1^), respectively. After 24 h, blood samples of the mice were collected in clean heparinized tubes for biochemical analysis. For the pharmacokinetic study of CAMNSN@PSN38, female BALB/c mice (n=5) were intravenously injected with CAMNSN@PSN38 (10 mg kg^-1^ of SN38) via tail veins. At predetermined time intervals (0, 0.5, 1, 2, 4, 6, 8, 12, and 24 h), blood samples (50 µL) of mice receiving treatments were collected from the orbital vein and mixed with NaOH (50 µL 0.1 M), they were then incubated at 45 °C for 12 h. Then acetonitrile (900 µL) was added and then it was ultrasonically dispersed and centrifuged to remove proteins. Finally, the supernatant (200 µL) was acidified with HCl (200 µL 0.1 M) before HPLC analysis. The concentrations of SN38 in blood were detected by HPLC using a method similar to that described above.

### Biodistribution of SN38 *in vivo*

Balb/c mice (n=5) were sacrificed 24 h after the intravenous injection of CPT-11 and CAMNSN@PSN38 ([SN38] = 10 mg kg^-1^). The major organs and tumor tissues were collected, homogenized, and mixed with NaOH (0.1 M) overnight at 37 °C, then the acetonitrile was added. The samples were centrifuged at 14000 rpm for 5 min, the supernatant was mixed with HCl (0.1 M) and analyzed *via* HPLC.

### Biodistribution of CAMNSN@PSN38 *in vivo*

The CT26 tumor-bearing mice were divided into 3 groups (n =3) and treated with intravenous injection of FITC-labeled CAMNSN@PSN38 (at a final e.q. [FITC] = 50 µg Kg^-1^) *via* the tail vein. The major organs and tumor tissues were collected and imaged by an IVIS^®^ Spectrum *in vivo* imaging system (PerkinElmer). Then, the major organs and tumor tissues were homogenized with PBS (pH = 7.4) plus Tween 80 (0.5 %), and homogenate obtained was analyzed *via* microplate reader by measuring the fluorescence of FITC.

### Statistical Analysis

Statistical analysis was performed using a two-sided unpaired Student's t-test through Excel. Data are expressed as mean ± SD.

## Results and Discussion

### Preparation and Characterization of CAMNSN@PSN38

**Figure 1:**
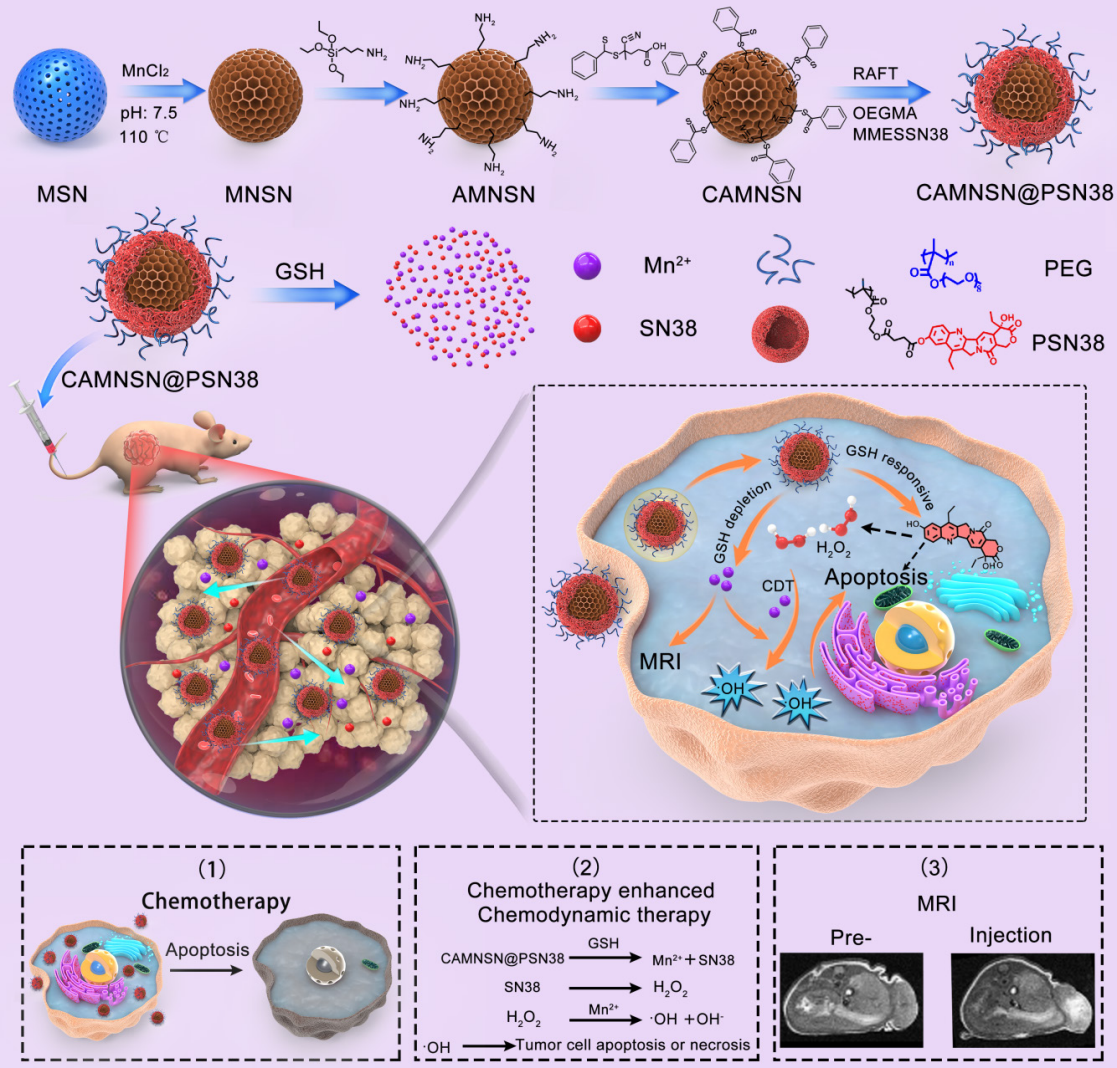
** Schematic of the process of fabricating CAMNSN@PSN38 and the therapeutic mechanism of chemodynamic/chemical synergistic therapy.** The biodegradable MnSiO_3_ nanosystem was synthesized step by step and the surface was modified with a reversible addition-fragmentation chain transfer (RAFT) chain transfer agent. The surface-initiated living radical polymerization of MMESSN38 (monomer of 7-Ethyl-10-hydroxycamptothecin (SN38)) and oligo(ethylene glycol) methacrylate (OEGMA) was conducted to obtain the final product, CAMNSN@PSN38. The nanoparticles delivered to the tumor tissues could be gradually biodegraded with glutathione (GSH), thus releasing SN38 and Mn^2+^ gradually. The SN38 released not only showed excellent chemical therapy effects but also improved the generation of H_2_O_2_. The interaction between CAMNSN@PSN38 and GSH consumes the GSH in tumor tissues, thereby releasing magnetic resonance imaging (MRI) and Fenton-like dual-functional agent Mn^2+^ for chemodynamic therapy and MRI.

The synthesis of CAMNSN@PSN38 is detailed in **Figure 1A**. First, well-dispersed mesoporous silica nanoparticles (MSNs) with an average diameter of about 100 nm were synthesized by a hydrothermal method. Hollow MnSiO_3_ nanoparticles (MNSNs) were then manufactured based on the well-dispersed MSNs. To carry out RAFT polymerization on the surface of MNSNs, aminopropyl groups were modified onto the exterior surface of the MNSN particles, followed by a reaction with 4-cyano-4-(thiobenzoylthio)pentanoic acid to attach chain transfer agent (CTA) groups. Thus, CAMNSN was obtained and ready for RAFT polymerization with the monomer of SN38. The CTA group of CAMNSN served in this role for the RAFT polymerization of OEGMA (Mw = 500) and MMESSN38 on the CAMNSN surface. After this, the CAMNSN@PSN38 nanoparticles were isolated and purified by dialysis and lyophilization. Thus, these well-engineered nanoparticles could be used as chemodynamic/chemical synergistic therapy agents and in MRI-guided combinatorial cancer therapy (**Figure 1B**).

**Figure 2:**
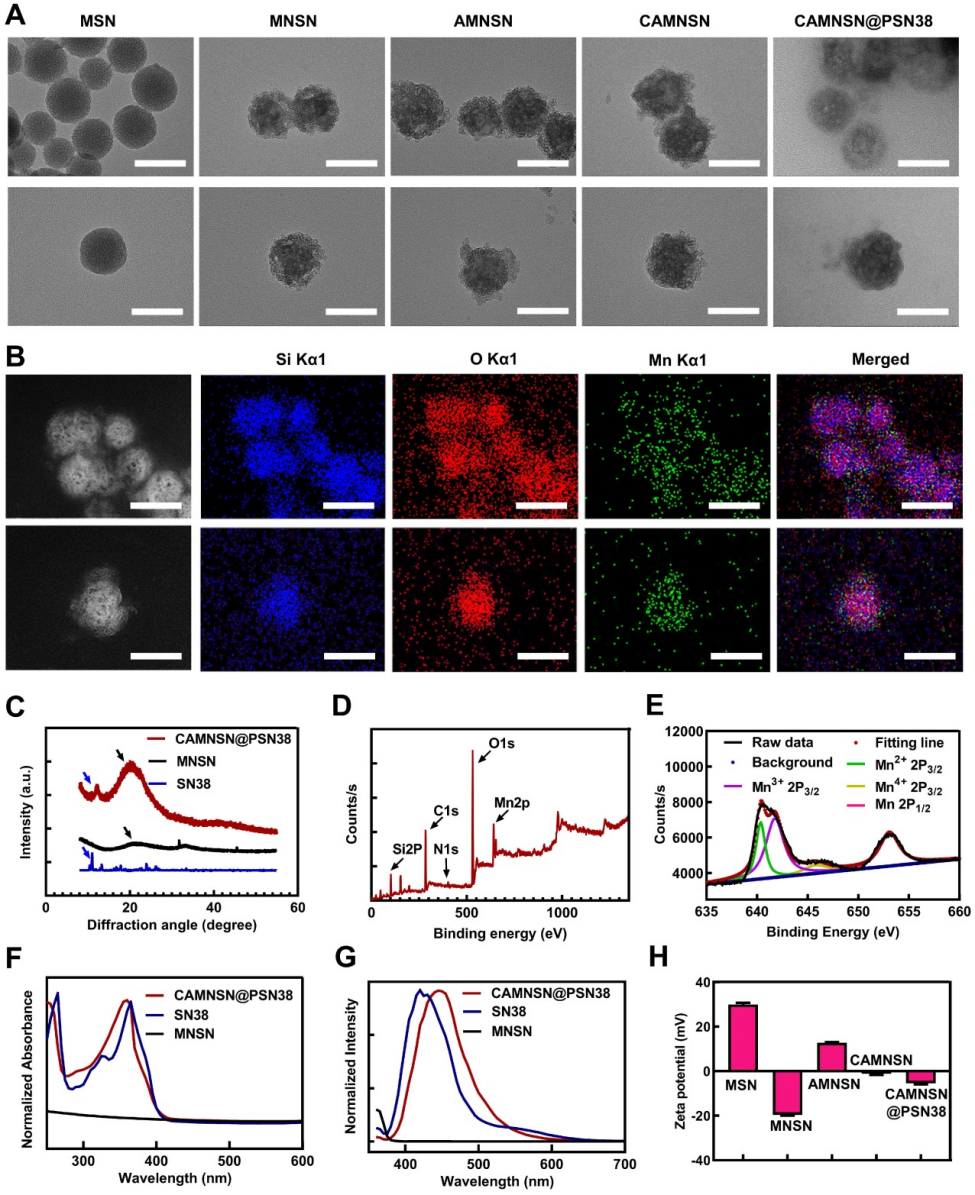
** Physiochemical characterizations. (A)** Transmission electron microscopy images of MSN, MNSN, AMNSN, CAMNSN and CAMNSN@PSN38. **(B)** Elemental mapping images of CAMNSN@PSN38. **(C)** X-ray diffraction spectra of MNSN, SN38 and CAMNSN@PSN38. **(D)** and** (E)** Full X-ray photoelectron spectra and high-resolution Mn2p XPS spectra of CAMNSN@PSN38. **(F)** and **(G)** Absorption spectra and fluorescence spectrum of CAMNSN@PSN38, SN38 and MNSN. **(H)** Zeta potential of MSN, MNSN, AMNSN, CAMNSN, and CAMNSN@PSN38. Scale bar: 100 nm.

As shown in the transmission electron microscopy (TEM) images, MSN, MNSN, AMNSN and CAMNSN with uniform size were synthesized step by step, then CAMNSN@PSN38 nanoparticles with an average diameter of 107.38 nm were synthesized finally (**Figure 2A**). The particle size increased slightly with the ligand integrating because small molecule layers and polymer layers are low contrast in TEM images. The hydrodynamic size of MSN and CAMNSN@PSN38 were measured by DLS, the results showed that the hydrodynamic size of CAMNSN increased significantly from 137.50 nm (MSN, PDI = 0.35) to 199.50 nm (PDI = 0.36). Furthermore, the stability of the CAMNSN@PSN38 was evaluated in the phosphate buffer solution (pH = 7.4), DLS results indicated that CAMNSN@PSN38 had good colloidal stability with the original size changed slightly over 1 week (**Figure S1**).

In addition, elemental mapping images of CAMNSN@PSN38 showed successful etching of the Mn element of the nanoparticles (**Figure 2B**). The polymerization of SN38 on the surface of MNSN was confirmed by X-ray diffraction (XRD) patterns with the characteristic peaks belonging to MNSN and SN38 (**Figure 2C**). According to the full spectra (**Figure 2D**) obtained by X-ray photoelectron spectroscopy (XPS), CAMNSN@PSN38 showed peaks assigned to Si, C, O, and Mn. Notably, the unique peak of N1s confirmed the polymerization of SN38. The high-resolution XPS profiles of Mn (**Figure 2E**) and Mn 2p_3/2_ confirmed that CAMNSN@PSN38 mainly consisted of Mn^2+^ (29.26%, 641 eV), Mn^3+^ (59.12%, 642 eV), and Mn^4+^ (11.62%, 644 eV). The relatively high concentration of Mn^3+^ in CAMNSN@PSN38 ensured that nanoparticles could biodegrade by reacting with endogenous GSH, thus releasing Mn^2+^ for CDT and MRI 36. Furthermore, the biodegradation of CAMNSN@PSN38 could deplete the ROS scavenger GSH, therefore amplifying the accumulation and therapeutic effect of ROS 37.

Subsequently, the composition and modification of the nanosystem were further characterized by UV-vis and fluorescence spectroscopies (**Figure 2F and G**). Compared with MNSN, CAMNSN@PSN38 featured a unique absorbance peak at 360 nm, demonstrating the presence of SN38. In addition, the fluorescence spectra of MNSN, SN38, and CAMNSN@PSN38 excited at a wavelength of 360 nm showed that the emission peak of CAMNSN@PSN38 at 440 nm was similar to that of SN38 at 420 nm. The phenolic hydroxyl ester bond between SN38 and the linker MMESCOOH (4-[2-(Methacryloyloxy)ethoxy]-4-oxobutanoic acid) was unstable. Alkaline hydrolysis in 0.1 M NaOH solution for 12 h at 40 °C was conducted to analyze the content of SN38 polymerized in CAMNSN@PSN38. The results of high-performance liquid chromatography (HPLC) analysis showed that the loading content of SN38, which was polymerized in our final product was 11.58% (wt%). Furthermore, thermogravimetric analysis (TGA) **(Figure S2)** was used to analyze the loss of organic weight in CAMNSN@PSN38. The results demonstrated that the content of polyethylene glycol was 77.62% and that of SN38 was 11.28% (MMESSN38: 17.30%); which was close to the results from the HPLC analysis. The zeta potential of MNSN was -19.67 mV while that of AMNSN was +12.73 mV, which proved the decoration of positively charged aminopropyl groups. The surface potentials of CAMNSN and CAMNSN@PSN38 were -1.11 mV and -5.48 mV, respectively, which was beneficial for the circulation and reduction of protein adsorption in blood.

SN38 is a potent drug that inhibits the growth of various tumor cells effectively 38, but the solubility of SN38 is extremely poor with a water solubility of 11 μg/mL 39. Moreover, SN38 has a strong crystallization tendency because of the strong π-π interaction among the SN38 molecules 40, resulting in the difficulty of delivering SN38. Surface-initiated living radical polymerization provides an inventive way to deliver drugs. Several studies reported surface-modified gold nanoparticles fabricated with living radical polymerization of the drugs recently 41, 42, which delivered drugs effectively without influence the function of Au nanoparticles. Currently, there are no reports that utilized the RAFT polymerization technique to produce polymers on the surface to modify the MNSN or MSN. The CAMNSN@PSN38 we synthesized through the living radical polymerization reaction in the surface of MNSN delivered SN38 effectively with the solubility of SN38 improved about 100 times, and avoided the influence of strong crystallization tendency of SN38.

The mesopores and surface areas of the nanoparticles were measured with the nitrogen adsorption-desorption experiment. The adsorption-desorption isotherm curves of MNSN, CAMNSN and CAMNSN@PSN38 demonstrated that compared with the surface area of MNSN (216.52 m^2^/g), that of CAMNSN (198.14 m^2^/g) remained unchanged. While the surface area of CAMNSN@PSN38 (22.35 m^2^/g) decreased significantly (**Figure S3A**). The corresponding pore size of MNSN and CAMNSN was 3.81 nm and 3.85 nm respectively, and the pore size of CAMNSN@PSN38 decreased to 3.01 nm (**Figure S3B**). These results suggested that the surface-initiated living radical polymerization of SN38 was succeeded and the polymer layer could obstruct the pore channels of MNSN effectively.

### GSH-Responsive Biodegradable Behavior of CAMNSN@PSN38

**Figure 3:**
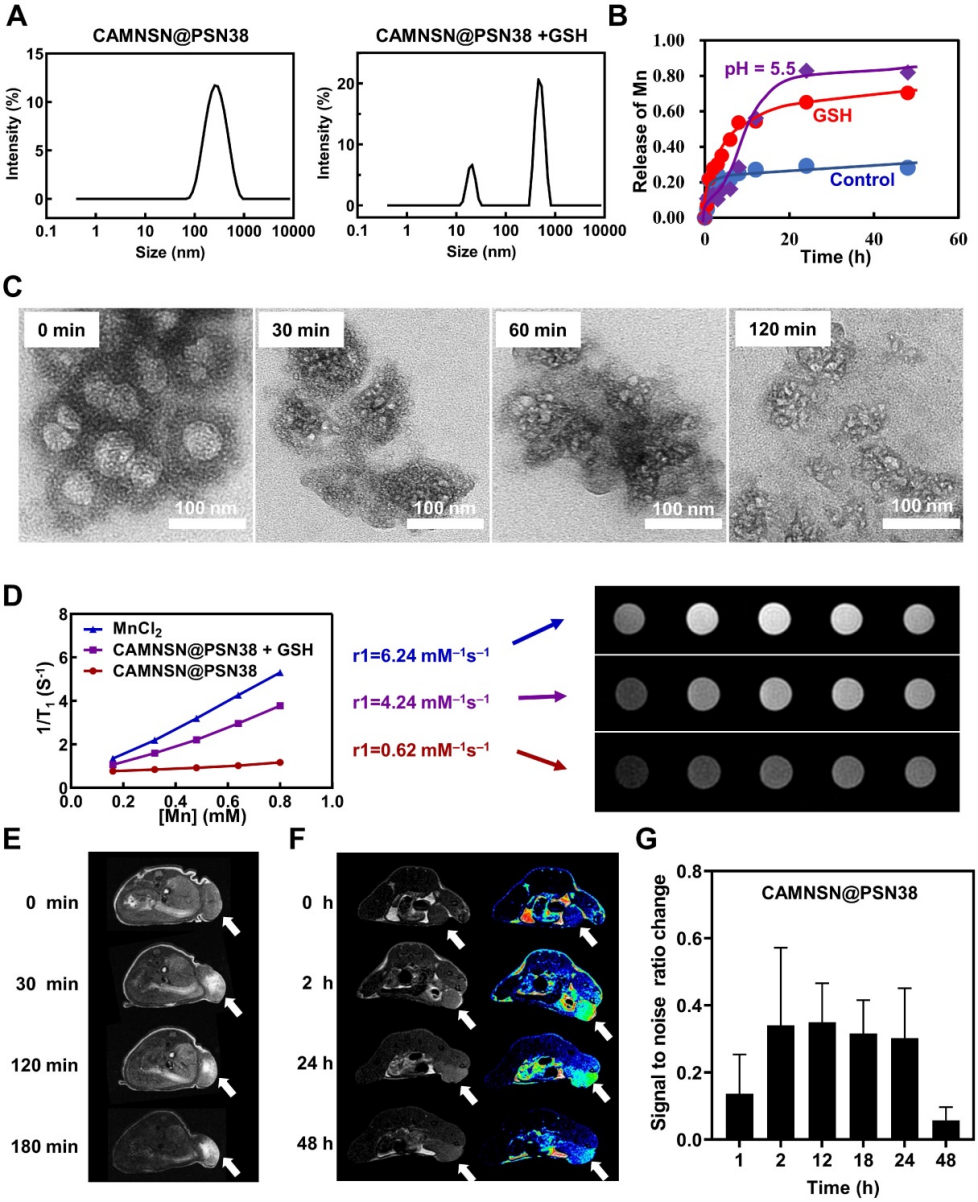
** Glutathione-responsive properties and magnetic resonance images of CAMNSN@PSN38 nanoparticles. (A)** The size distribution of MSN, CAMNSN, CAMNSN@PSN38 nanoparticles treated with or without GSH (10 mM). **(B)** The release curve of CAMNSN@PSN38 treated with 10 mM GSH or mildly acidic solution (pH = 5.5). **(C)** TEM images of CAMNSN@PSN38 nanoparticles treated with 10 mM GSH at 0 min, 30 min, 60 min and 120 min. **(D)** *In vitro* MRI of MnCl_2,_ CAMNSN@PSN38 nanoparticles treated with or without GSH (10 mM), and the corresponding r1 value. *In vivo* T1-weighted MR imaging of CT-26 tumor-bearing mice treated with CAMNSN@PSN38 nanoparticles (e.q. [SN38]: 10 mg/kg) at different time points after intratumor injection **(E)** or injection through the tail vein **(F)**. **(G)** Quantitative detection of the signal-to-noise ratio change in the T1-weighted MR image of the tumor site at different time points. Arrow: tumor tissues.

It is an effective and innovative strategy to construct a tumor microenvironment responsive nanosystem by rational design 43, 44. Several studies have manifested that tumor microenvironment responsive nanosystem based on the specific physiological features of colorectal cancer offers a paradigm in the synergistic treatment of colorectal cancer 45, 46. As previously reported 36, Mn-O bonds enriched in MNSN can be cracked by GSH as shown by TEM, resulting in the release of Mn^2+^. Additionally, the phenol ester bond in the SN38 monomer was weak; the ester bond could also be disrupted by GSH, thus the release rate of SN38 was accelerated in the presence of GSH 47. The CAMNSN@PSN38 consisted of GSH-depleted MNSN and GSH-responsive phenol ester bonds, which could be realized as enormous potential “turn-on” therapeutic tools for the targeted and efficient treatment of colorectal cancer. Dynamic light scattering showed that the distribution of CAMNSN@PSN38, in terms of hydrodynamic size, was divided into two peaks with the addition of GSH (**Figure 3A**). The TEM images further confirmed that the structure of CAMNSN@PSN38 could be biodegraded over time with the addition of GSH (**Figure 3C**). These results suggest that the CAMNSN@PSN38 could be biodegraded under the trigger of GSH. Furthermore, the tumor microenvironment responsive property of CAMNSN was not influenced by the surface-initiated living radical polymerization.

The release rate of Mn^2+^ increased from 28 % to 70 % with the addition of GSH. Interestingly, the release of Mn^2+^ also accelerated in the mild acidosis environment (pH = 5.5) with the release rate increased to 82% (**Figure 3B**). Furthermore, the release of SN38 was also increased with the addition of GSH, the release rate of SN38 increased from 13 % to 54 % at 48 h with the concentration of GSH increased to 10 mM **(Figure S4)**. The drug could be loaded in the pores of MNSN, but the leaking of the drug was about 40 % in 24 h 30. The phenol ester bond between SN38 and the polymer in the surface of MNSN prevents the SN38 from leaking with only 13 % of SN38 released at 48 h.

The Mn^2+^ released from CAMNSN@PSN38 was a great MR contrast agent. As shown in** Figure 3D**, the T1 signal intensity of CAMNSN@PSN38 changed negligibly with r1 = 0.62 mM^-1^S^-1^. After incubation with GSH, the CAMNSN@PSN38 + GSH group exhibited enhanced brightness, with r1 increasing to 4.24 mM^-1^S^-1^ owing to the GSH-induced reduction of CAMNSN@PSN38. The r1 value for the CAMNSN@PSN38 + GSH group was close to that of free Mn^2+^ (r1 = 6.24 mM^-1^S^-1^). The signal intensity appears to increase first and then decrease slightly because what we showed in the manuscript were magnitude images. The T1-weighted MR scan was carried out with an inversion recovery (IR) pulse sequence, from which the contrast behavior can be very different in magnitude and real (or phase-sensitive IR) images. Although the software at our scanner exports only the magnitude images, it utilizes real (phase-sensitive) images in calculating the r1 values. Therefore, the r1 value obtained was not influenced.

At the same time, we investigated the MRI properties of CAMNSN@PSN38 injected intratumor or through the tail vein *in vivo*. As shown in **Figure 3E**, the brightness of tumor tissues in mice treated with CAMNSN@PSN38 was enhanced over time, indicating that CAMNSN@PSN38 could be disrupted into Mn^2+^ by GSH in tumor tissues and the Mn^2+^ released was a proper MRI contrast agent. Furthermore, we investigate the MRI property through the tail vein injection of CAMNSN@PSN38 (**Figure 3F**). It could be seen that the MR image at 2 h post-injection was much brighter and the bright tumor region was maintained over 24 h after injection. In addition, the signal-to-noise ratio change in the tumor region of MR images were analyzed (**Figure 3G**). The results indicated that the signal-to-noise ratio change increased gradually at 1 h (14%) and maintained a high level in 2 h (34%), 12 h (35%), 18 h (32%) and 24 h (30%). The results suggested that the CAMNSN@PSN38 nanoparticles were accumulated in the tumor tissues and the Mn^2+^ was controlled released under the tumor microenvironment. The great MRI property of CAMNSN@PSN38 provided a useful tool for guiding and monitoring the therapy of CRC.

### Intracellular Uptake of CAMNSN@PSN38

**Figure 4:**
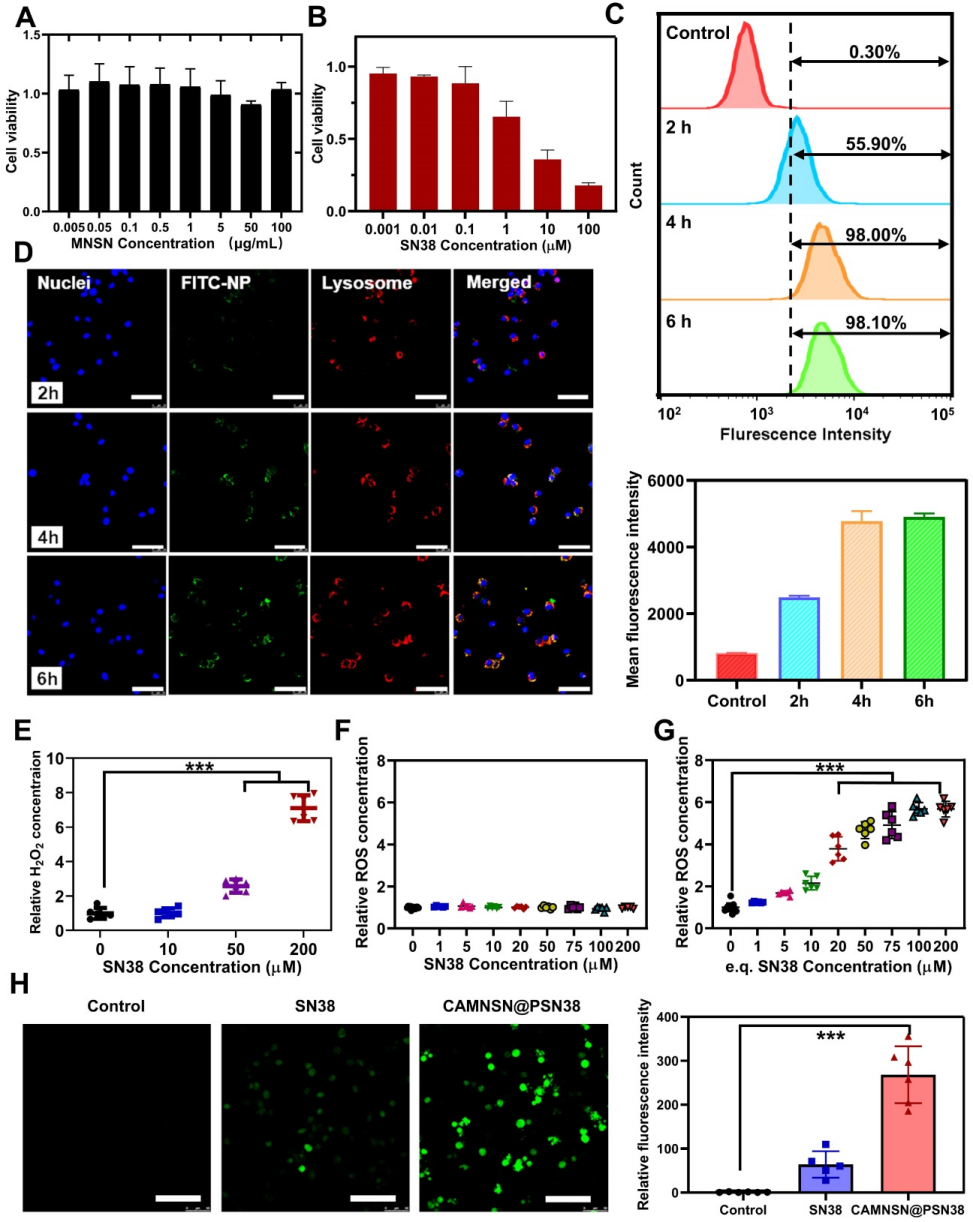
** Intracellular uptake and ROS generation of CAMNSN@PSN38. (A& B)** Cell viability of CT26 cells treated with different concentrations of MNSN and CAMNSN@PSN38 by cell counting kit-8 assay. Data are presented as mean ± SD (n = 3). **(C)** The rate of uptake of CAMNSN@PSN38 into CT26 cells and the corresponding mean fluorescence intensity by flow cytometry after different incubation times. **(D)** Fluorescence images of CT26 cells showing cellular uptake of CAMNSN@PSN38 after incubation for 2, 4, and 6 h. Scale bar: 100 µm. **(E)** The levels of H_2_O_2_ were measured with Amplex Red Peroxide assay after SN38 treatment in CT26 cells. **(F & G)** The levels of reactive oxygen species (ROS) were measured with dichloro-dihydro-fluorescein diacetate after SN38 and CAMNSN@PSN38 treatment in CT26 cells. **(H)** Fluorescence images and corresponding mean fluorescence intensity showing ROS levels in CT26 cells with the treatment of CAMNSN@PSN38 (e.q. [SN38] = 20 µM). Scale bar: 100 µm, mean ± SD, ***p < 0.001.

Before evaluating the anti-tumor efficiency of CAMNSN@PSN38 *in vivo*, the cytotoxicity and cell uptake of MNSN and CAMNSN@PSN38 were investigated. MNSN showed less cytotoxicity towards CRC cell lines including CT26, HT29, and SW620 after 48 h incubation (**Figure 4A, S5 and S6**), indicating good biocompatibility of MNSN. However, the cytotoxicity of CAMNSN@PSN38 was enhanced by the polymerization of SN38 (**Figure 4B**). After this, the fluorescein isothiocyanate (FITC) antigen was conjugated onto the surface of CAMNSN@PSN38 to trace the nanoparticles. To analyze the cell uptake of CAMNSN@PSN38, flow cytometry was used to measure the fluorescence intensity of FITC in CT26 cells treated with CAMNSN^-FITC^@PSN38 at 0, 2, 4, and 6 h (**Figure 4C**). This FITC analysis was shown as mean fluorescence intensity in** Figure 4C**. The CAMNSN^-FITC^@PSN38 treatment group showed a relatively high uptake rate compared to the control group. As the incubation time increased, the uptake rate of the CAMNSN^-FITC^@PSN38 group increased from a base level of 55.90% to 98.00% in 2 h, moreover, it increased up to 98.10% at 4 and 6 h. Fluorescence images in **Figure 4D** demonstrate similar cell uptake behavior of CAMNSN^-FITC^@PSN38. Furthermore, after incubation for 2, 4, and 6 h, the fluorescence location of CAMNSN^-FITC^@PSN38 in CT26 cells overlapped with that of the lysosome, suggesting that the uptake was *via* the endolysosomal pathway (**Figure 4D and S7**). The release of Mn^2+^ also accelerated in the mild acidosis environment **(Figure 3B)**, suggesting that the CAMNSN@PSN38 was sensitive to the mildly acidic solution. The environment of the intracellular stroma of cancer cells was mild acidosis 48 and the pH in the lysosome was about 5.5. The sensitive property in the mildly acidic environment makes the CAMNSN@PSN38 a smart and controllable delivery system to deliver SN38 and Mn^2+^.

### ROS Production Improvement with CAMNSN@PSN38

The contents of the specific oxidant H_2_O_2_ were tested with the Amplex Red Peroxide Assay to determine whether the concentration of H_2_O_2_ could be influenced by SN38. Interestingly, as shown in **Figure 4E**, the production of H_2_O_2_ increased with the addition of SN38. However, the ROS content in CT26 cells was also analyzed (**Figure 4F**); ROS levels were not influenced by the increased SN38 concentration. Previous research demonstrated that ROS generation could be reduced by GSH produced in cancer cells 12. The presence of manganese silicate in CAMNSN@PSN38 could deplete GSH 16, 25. As shown in **Figure 4G**, ROS accumulation was detected with the ROS probe dichloro-dihydro-fluorescein diacetate (DCFH-DA). CAMNSN@PSN38 ROS generation increased markedly when compared to SN38 at the same concentrations. Furthermore, fluorescence imaging of DCFH-DA in CT26 cells demonstrated that the cells in the CAMNSN@PSN38 treated group displayed a much stronger green fluorescence than in the SN38 treated group, which had only a weak signal (**Figure 4H**). The corresponding analysis of the ROS probe fluorescence intensity was also performed. These results indicate that the generation and accumulation of ROS were enhanced by CAMNSN@PSN38 in the presence of manganese silicate and SN38.

Electron spin resonance (ESR) spectroscopy was applied to confirm the catalytic functionality of CAMNSN@PSN38 and the species of ROS. The detection was performed in a prepared mildly acidic H_2_O_2_ solution (pH = 5.5), which mimics the intracellular microenvironment with mild acidosis and redox adaptation. The lifetime of •OH was extremely short (10^-9^ s), so 5,5-dimethyl-1-pyrroline-N-oxide (DMPO) was used as a nitrone spin trap to trap the forming •OH. Characteristic 1:2:2:1 signal of •OH was detected in the ESR spectrum of CAMNSN@PSN38 in the mildly acidic H_2_O_2_ solution, while no signal was detected in the acidic solution absence of H_2_O_2_ (**Figure S8**), demonstrating the catalytic activity of CAMNSN@PSN38 was high and the species of ROS was hydroxyl radicals.

### *In vivo* Anti-Tumor Efficiency of CAMNSN@PSN38 in a Transplanted Subcutaneous Tumor Model

**Figure 5:**
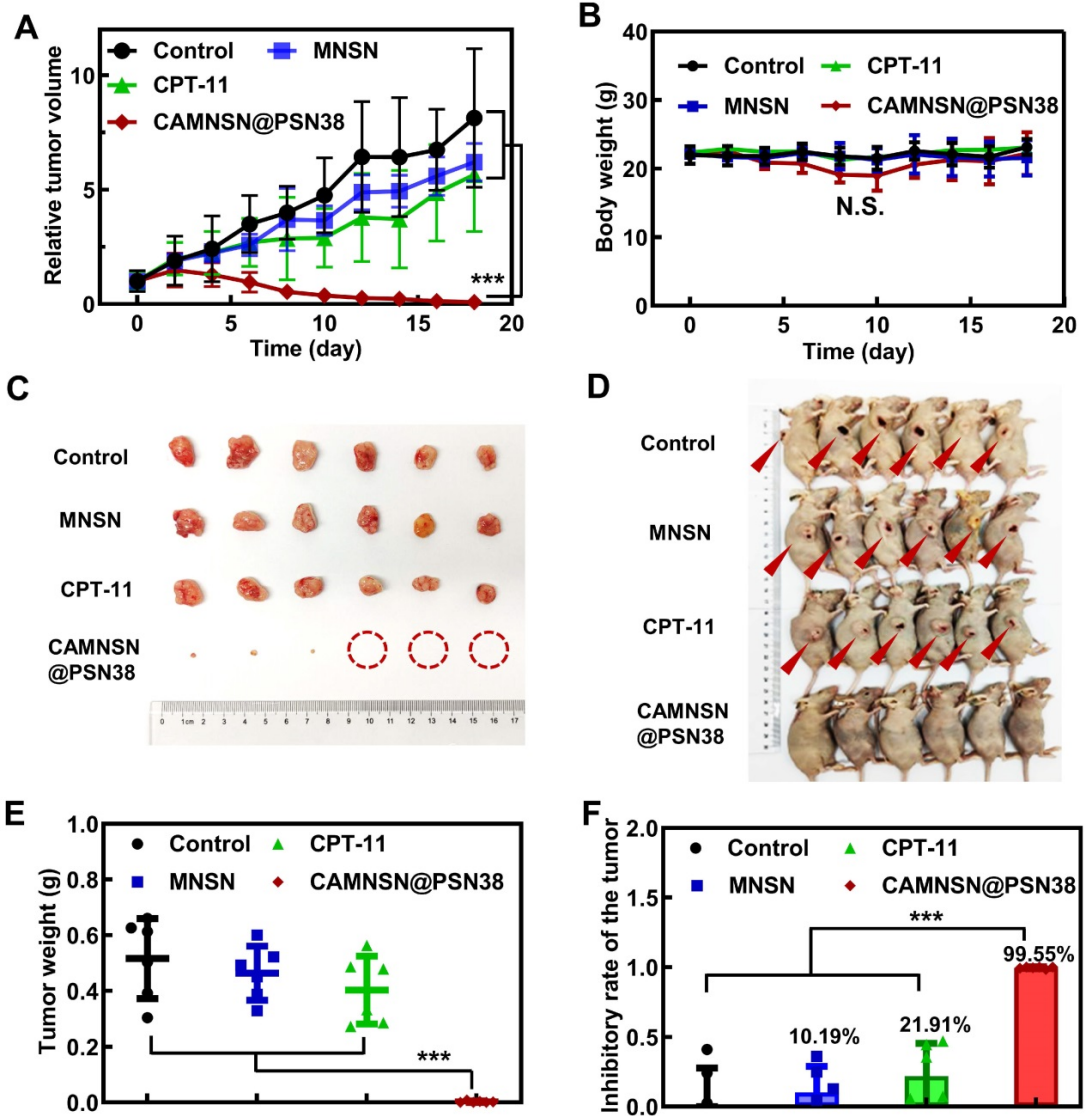
** Antitumor activity of CAMNSN@PSN38 on the HT29 tumor model. (A)** Antitumor activity of CAMNSN@PSN38 on HT29 tumor models in terms of tumor volume as a function of time. **(B)** The systemic toxicity of CAMNSN@PSN38 was evaluated by body weight changes with the treatments. **(C)** Images of the HT29 tumor excised at the end of the experiments. **(D)** Photos of the mice with different treatments at the end of the experiments. **(E and F)** Tumor weight and corresponding inhibitory rate of the tumor of each group at the end of the experiment. Balb/c nude mice bearing HT29 xenograft tumors were treated with CPT-11 (e.q. SN38 concentration: 10 mg kg^-1^), MNSN (5 mg kg^-1^), and CAMNSN@PSN38 (e.q. SN38 concentration: 10 mg kg^-1^, e.q. MNSN concentration: 5 mg kg^-1^) with tail vein injection every other day 3 times over. n = 5, mean ± SD, ***p < 0.001.

The tumor treatment efficacy of CAMNSN@PSN38-mediated chemodynamic/chemical synergistic therapy* in vivo* was first identified in the HT29 transplant subcutaneous tumor model. As shown in **Figure 5A**, treatment with either MNSN (5 mg kg^-1^) or CPT-11 (10 mg kg^-1^) alone by intravenous administration showed little anti-tumor activity. Remarkably, CAMNSN@PSN38 (e.q. MNSN con. = 5 mg kg^-1^, e.q. CPT-11 con. = 10 mg kg^-1^) showed a markedly greater anti-tumor efficacy than MNSN and CPT-11. The tumor tissues in the CAMNSN@PSN38 treatment group gradually shrank and almost disappeared after three injections (**Figure 5C and 5D**). The results of tumor weight measurements (**Figure 5E and 5F**) 14 days after the final treatment demonstrated that CAMNSN@PSN38 had a 99.55% tumor inhibition, markedly higher than for MNSN (10.19%) and CPT-11 (21.91%). The bodyweight of mice treated with CAMNSN@PSN38 decreased slightly, however, once the treatment was terminated, they returned to normal (**Figure 5B**).

**Figure 6:**
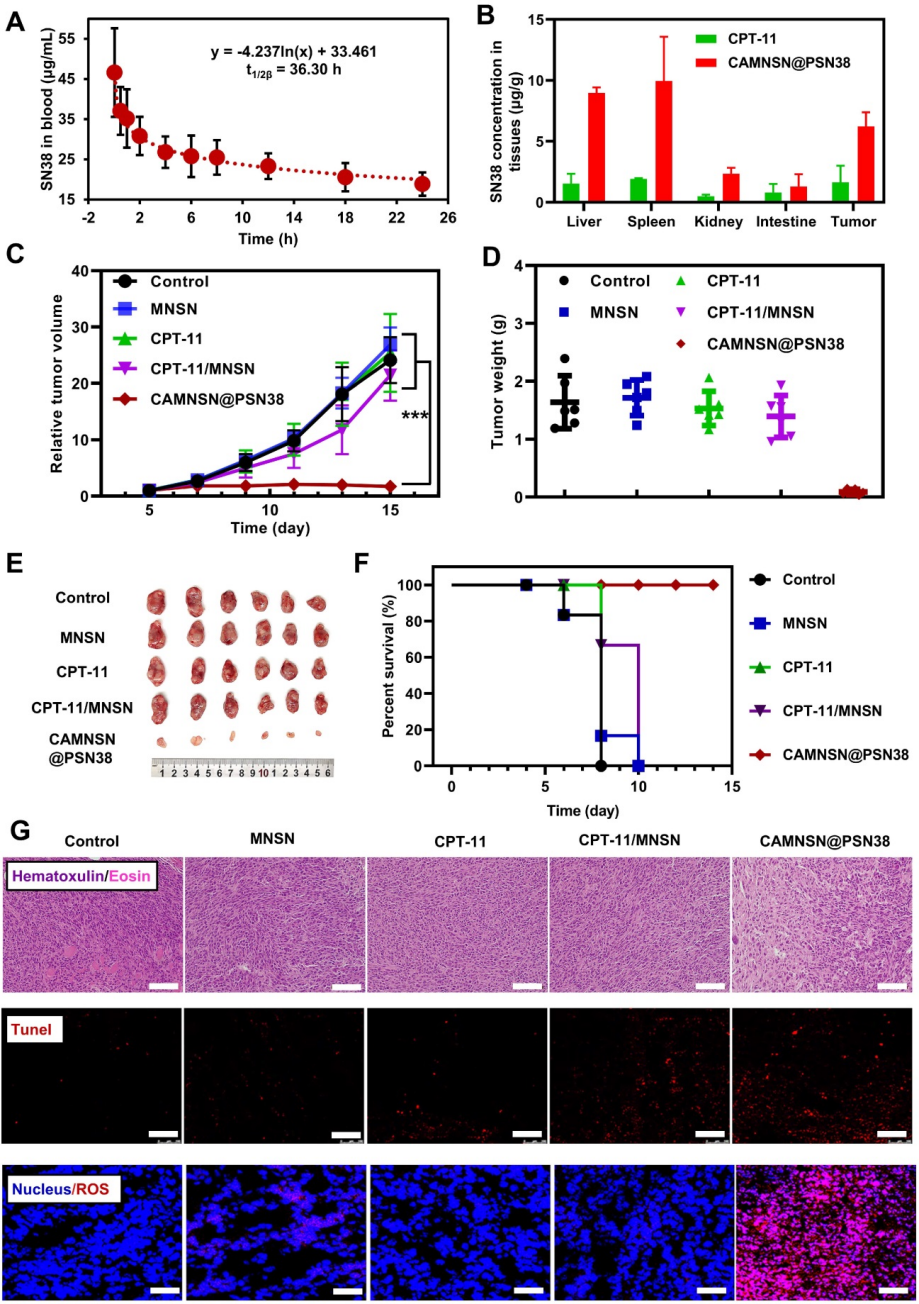
** Antitumor activity and chemodynamic/chemical synergistic therapy of CAMNSN@PSN38 on the CT26 tumor model. (A)** The blood circulation of CAMNSN@PSN38 via SN38 concentration measurement for 24 h after intravenous injection. Data are presented as mean ± SD (n = 3) and **(B)** biodistribution of CPT-11 and CAMNSN@PSN38 by measuring SN38 concentrations at 24 h after intravenous injection. Data are presented as mean ± SD (n = 3). **(C)** Antitumor activity of CAMNSN@PSN38 on CT26 tumor models in terms of tumor volume as a function of time. **(D)** Tumor weights of each group at the end of the experiment. **(E)** Images of the CT26 tumor excised at the end of the experiments. **(F)** Photos of the mice with different treatments at the end of the experiments. **(F)** Survival ratio of the mice bearing CT26 tumors treated with different treatments. **(G)** Hematoxylin and eosin -, terminal deoxynucleotidyl transferase dUTP nick end labeling -, and reactive oxygen species stained tumor slices collected from different treatments at the end of the experiment. Scale bar: 100 μm. Balb/c nude mice bearing CT26 xenograft tumors were treated with CPT-11 (e.q. SN38 concentration: 10 mg kg^-1^), MNSN (5 mg kg^-1^), CPT-11/MNSN (e.q. SN38 concentration: 10 mg kg^-1^, e.q. MNSN concentration: 5 mg kg^-1^) and CAMNSN@PSN38 (e.q. SN38 concentration: 10 mg kg^-1^, e.q. MNSN concentration: 5 mg kg^-1^) with tail vein injection every other day 3 times over. n = 5, mean ± SD, ***p < 0.001.

Furthermore, the tumor treatment efficacy of chemodynamic/chemical synergistic therapy* in vivo* was analyzed in the CT26 cell line transplanted into the subcutaneous tumor model. As shown in **Figure 6A**, the blood circulation behavior of CAMNSN@PSN38 was measured by detecting the concentrations of SN38 in the blood at different time points. A long circulation time of SN38 was observed, which was favorable for effective CAMNSN@PSN38 accumulation in tumor tissues with the enhanced permeability and retention effect. The concentration of SN38 in the major organs of mice treated with CAMNSN@PSN38 and CPT-11 were also analyzed, as shown in **Figure 6B**. The concentration of SN38 in the CAMNSN@PSN38 treated group was higher than that of CPT-11, confirming the enhanced accumulation of CAMNSN@PSN38 with higher circulation time. The biodistribution of the CAMNSN@PSN38 *in vivo* was carefully evaluated by tracking the labeled FITC fluorescence in major organs and tumor tissues over time (**Figure S9**). CAMNSN@PSN38 was observed to accumulate in the liver, which could be attributed to the reticuloendothelial system. On the other hand, it could be observed that CAMNSN@PSN38 was effectively accumulated in the tumor, which can endow CAMNSN@PSN38 an excellent MR contrast agent property and a great therapy effect for tumor tissues. The pharmacokinetic behaviors and biodistribution of the CAMNSN@PSN38 *in vivo* were further investigated by quantifying the concentration of CAMNSN^-FITC^@PSN38. It could be seen that the highest concentration of CAMNSN@PSN38 in major organs and tumor tissues appeared at 8 h post-injection, which suggested that CAMNSN@PSN38 had a long retention time and the nanoparticles were accumulated in tumor tissues gradually. After 24 h, the content of CAMNSN@PSN38 in all major organs descended, suggesting that the CAMNSN@PSN38 could be gradually excreted from the body over time and the nanoparticles would not raise the risk of harm to the body.

Relatively high levels of nanoparticles were observed in the liver, spleen and kidney, so biosafety evaluation of CAMNSN@PSN38 was conducted. As shown in **Figure S10 and S11**, normal histology examinations (hematoxylin and eosin (H&E) staining) of the main organs, relatively stable blood biochemistry assays, and complete blood panel analyses further suggested the negligible side effects of the CAMNSN@PSN38 treatment in mice.

The anti-tumor efficacy of CAMNSN@PSN38 was further evaluated in CT26 tumor-bearing mice. Minimal inhibitory effects were observed in the MNSN and CPT-11 groups with the tumor growth similar to the control group (**Figure 6C**). However, the free drug combinations of CPT-11 and MNSN showed some tumor inhibition effects. Notably, tumor sizes were markedly suppressed in the CAMNSN@PSN38 treatment group after three injections with the chemodynamic/chemical synergistic therapy (**Figure 6D, 6E, and S12**). The survival curve also demonstrated the strong anti-tumor effect of CAMNSN@PSN38 (**Figure 6F**). The corresponding H&E and terminal deoxynucleotidyl transferase dUTP nick end labeling (TUNEL) stains on tumor tissues showed that the CAMNSN@PSN38 treated group achieved maximum tumor necrosis and apoptosis (Figure 6G and 6H). Furthermore, the dihydroethidium staining was used to show the ROS contents in tumor tissues. The red fluorescence of ROS indicated that the anti-tumor activity of CAMNSN@PSN38 was due to the strong generation of ROS in this therapy. These results indicated that the excellent anti-tumor efficiency of CAMNSN@PSN38 was due to the combined chemodynamic/chemical therapy.

### *In vivo* Tumors Suppress the Activity of CAMNSN@PSN38 in a Peritoneal Disseminated Tumor Model

**Figure 7:**
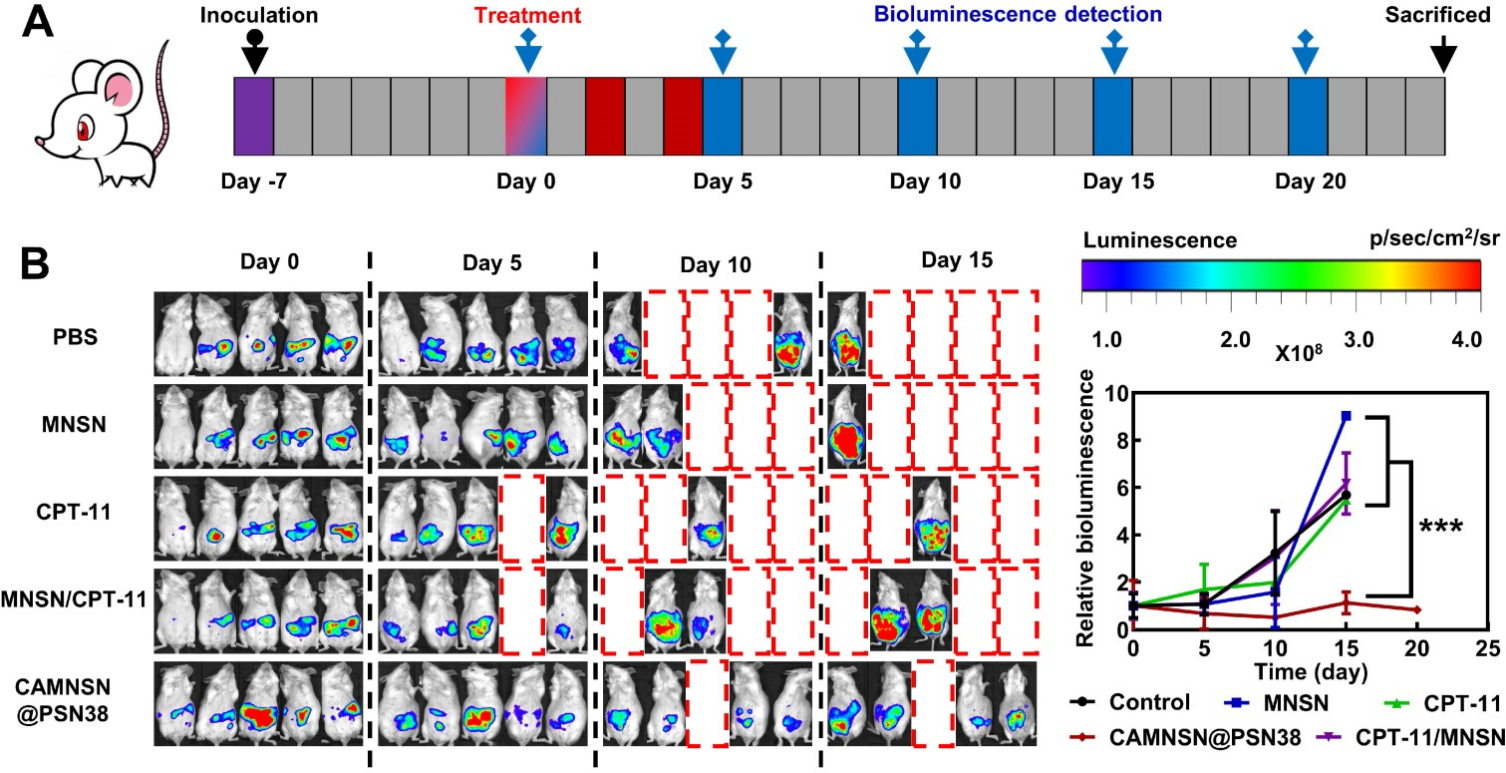
** CAMNSN@PSN38 showed superior antitumor activity in the intraperitoneal tumor model. (A)** Therapeutic and bioluminescence detection schedule for different treatments. **(B)** Tumor growth was detected *via* luciferase bioluminescence detection. Bioluminescence values were normalized with the value detected at day 0. Balb/c nude mice bearing CT26-luc intraperitoneal tumors were treated with CPT-11 (e.q. SN38 concentration: 10 mg kg^-1^), MNSN (5 mg kg^-1^), CPT-11/MNSN (e.q. SN38 concentration: 10 mg kg^-1^, e.q. MNSN concentration: 5 mg kg^-1^) and CAMNSN@PSN38 (e.q. SN38 concentration: 10 mg kg^-1^, e.q. MNSN concentration: 5 mg kg^-1^) with tail vein injection every other day 3 times over. N = 5, mean ± SD, ***p < 0.001.

To further examine the anti-tumor efficiency of CAMNSN@PSN38, a CT26-luc peritoneal disseminated tumor model was established and corresponding treatments were performed based on the therapeutic schedule shown in **Figure 7A**. Bioluminescence imaging showed that the growth of tumors in the control group was rapid. MNSN alone, CPT-11 alone, and MNSN/CPT-11 free drug combination treatments showed fewer effects on stopping the growth of the tumor (**Figure 7B**). Moreover, the peritoneal disseminated tumor model was quite lethal, and most mice died 5 days after the treatment. However, the bioluminescence signal of the CAMNSN@PSN38 treated group was low, moreover, the majority of the CAMNSN@PSN38 treated group were alive 10 days after the treatment (**Figure S13**).

## Conclusion

In conclusion, a CRC tumor microenvironment-responsive nanosystem was prepared for both chemodynamic/chemical synergistic therapy and MRI-guided therapy. The system was based on a hybrid nanomaterial, in which SN38 was grown *in situ* on the surface of MNSNs with RAFT surface-initiated living radical polymerization. This nanosystem, CAMNSN@PSN38, increased the cycling time and solubility of SN38. Interestingly, SN38 was able to improve the concentration of H_2_O_2_ but not the ROS content. We speculate that the GSH in tumor tissues consumed the increased H_2_O_2_ content. Notably, triggered by GSH, the CAMNSN@PSN38 delivered to the tumor tissues could gradually release SN38 and Mn^2+^ simultaneously. The MnSiO_3_ in the hybrid nanomaterial guaranteed the consumption of GSH and the accumulation and generation of H_2_O_2_. Owing to the release of Mn^2+^, the level of ROS also improved markedly. *In vivo* studies demonstrated excellent synergistic effects of the CAMNSN@PSN38-mediated therapy, which effectively suppressed the growth of tumors in three CRC tumor models, alongside the improved accumulation of ROS and controlled release of SN38. This work provided a promising paradigm for both the treatment and MRI-guided therapy of colorectal cancer, *via* the simultaneous release of the chemotherapeutic drug, GSH expenditure, and improved ROS generation.

## Supplementary Material

Supplementary figures and tables.

## Abbreviations

MRI: magnetic resonant imaging
GSH: glutathione
ROS: reactive oxygen species
CRC: Colorectal cancer
CDT: chemodynamic therapy
•OH: hydroxyl radicals
SN38: 7-ethyl-10-hydroxycamptothecin
RAFT: reversible addition-fragmentation chain transfer
MMESSN38: monomer of SN38
OEGMA: oligo(ethylene glycol) methacrylate
MSN: mesoporous silica nanoparticles
MNSN: MnSiO3 nanoparticles
AMNSN: Amino-modified MNSN
CAMNSN: RAFT chain transfer agent-modified MNSN
CAMNSN@PSN38: The polymerization product of OEGMA and MMESSN38 on CAMNSN
CLSM: Confocal laser scanning microscope
H&E staining: hematoxylin-eosin staining
TUNEL: Terminal deoxynucleotidyl transferase dUTP nick end labeling
DCFH-DA: 2,7-Dichlorodi -hydrofluorescein diacetate
DAPI: 4',6-diamidino-2-phenylindole
