# Supplementary material for: A tumor microenvironment responsive nanosystem for chemodynamic/chemical synergistic theranostics of colorectal cancer
Source: Theranostics. 2021 Aug 18;11(18):8909–25. doi: 10.7150/thno.61651 (PMC8419042; doi:10.7150/thno.61651)
Supplement: Supplementary file 1 — Supplementary figures and tables. [file thnov11p8909s1.pdf]

Supporting Information

A tumor microenvironment responsive nanosystem for chemodynamic/chemical synergistic theranostics of colorectal cancer

Liying Wang, Jingya Xia, Hongjie Fan, Min Hou, Huiyang Wang, Xiaoyan Wang, Ke Zhang, Liping Cao, Xiangrui Liu, Jun Ling, Hong Yu, Xia Wu*,Jihong Sun**

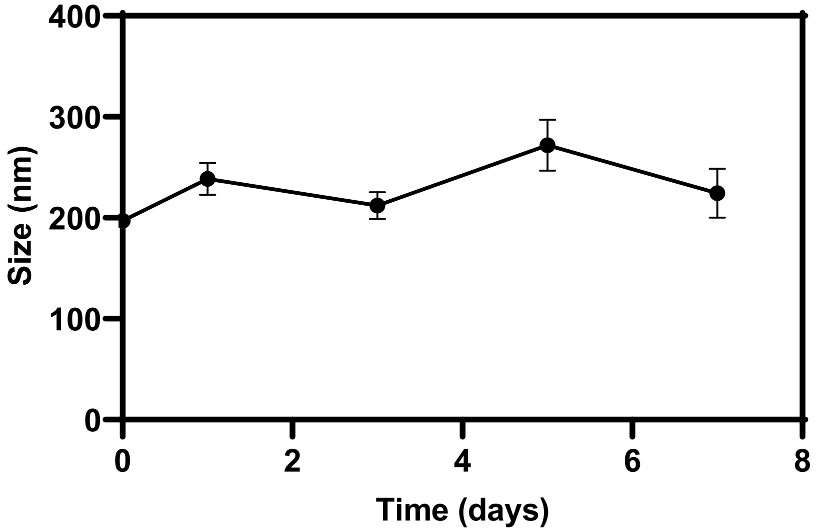

Figure S1. The size change of CAMNSN@PSN38 phosphate buffer solution (pH = 7.4).

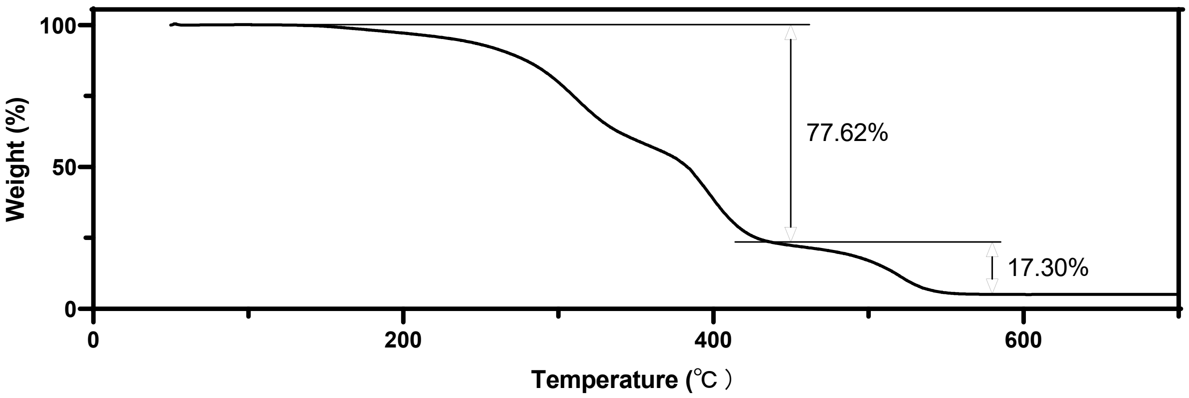

Figure S2. TGA curve of CAMNSN@PSN38.

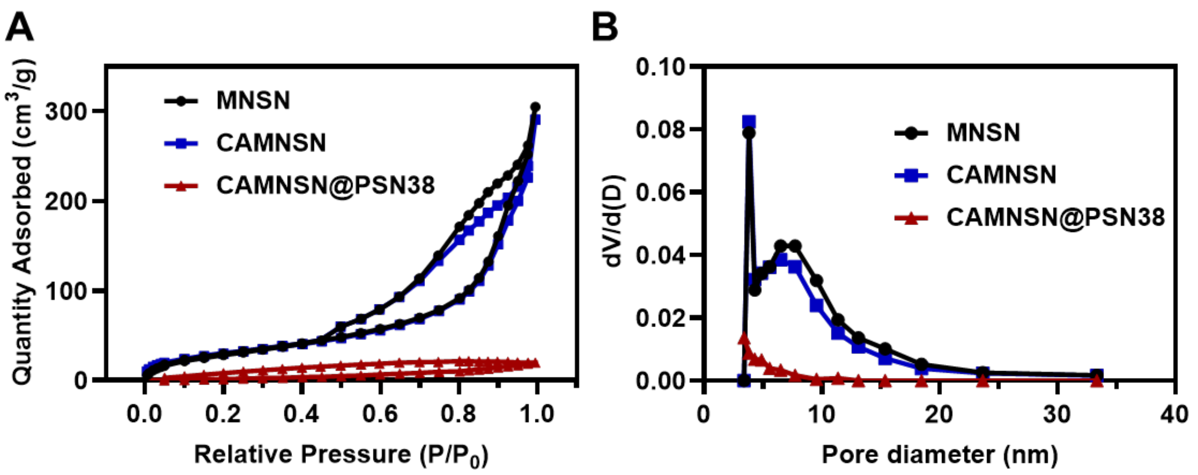

Figure S3. N2 adsorption/desorption isotherms (A) and pore size distribution (B) of MNSN, CAMNSN and CAMNSN@PSN38.

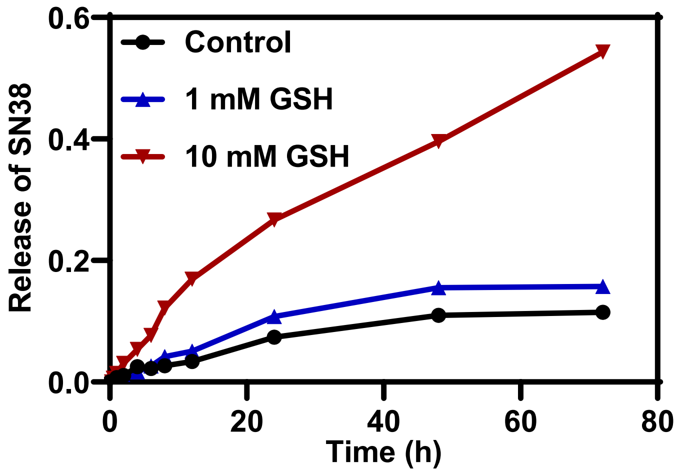

Figure S4. The release curve of SN38 at different time points after the addition of GSH.

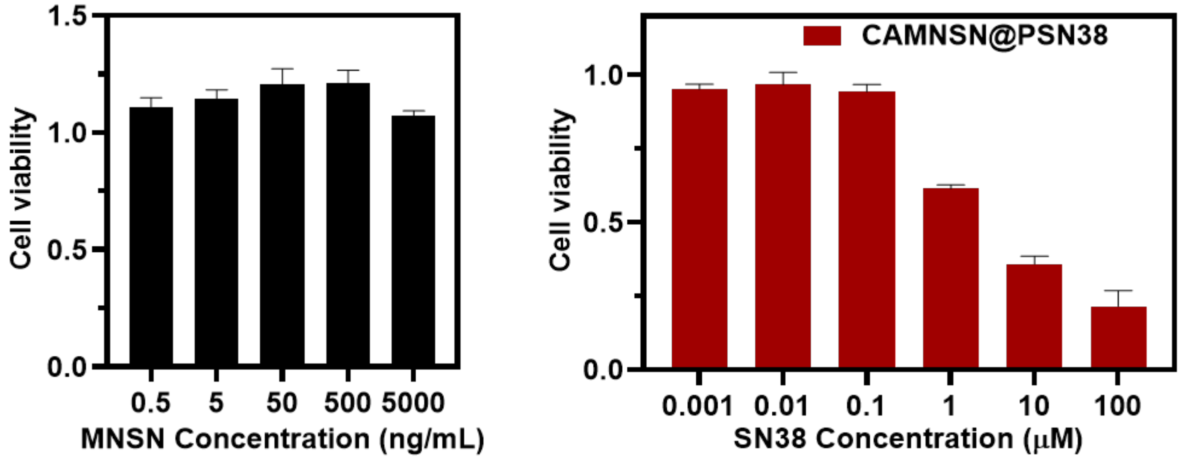

Figure S5. The cytotoxicity assay of MNSN and CAMNSN@PSN38 on the SW620 cell line was measured by CCK8 kits at 48 h.

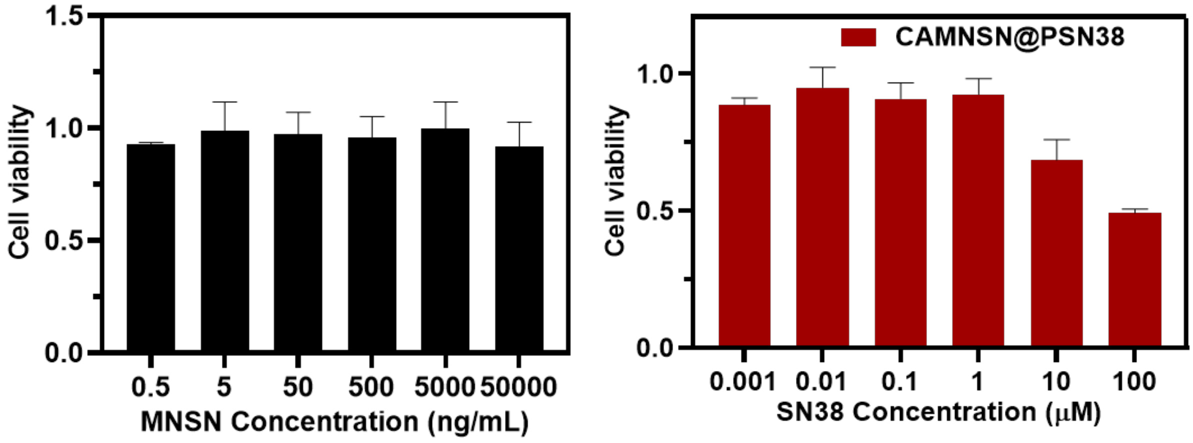

Figure S6. The cytotoxicity assay of MNSN and CAMNSN@PSN38 on the HT29 cell line was measured by CCK8 kits at 48 h.

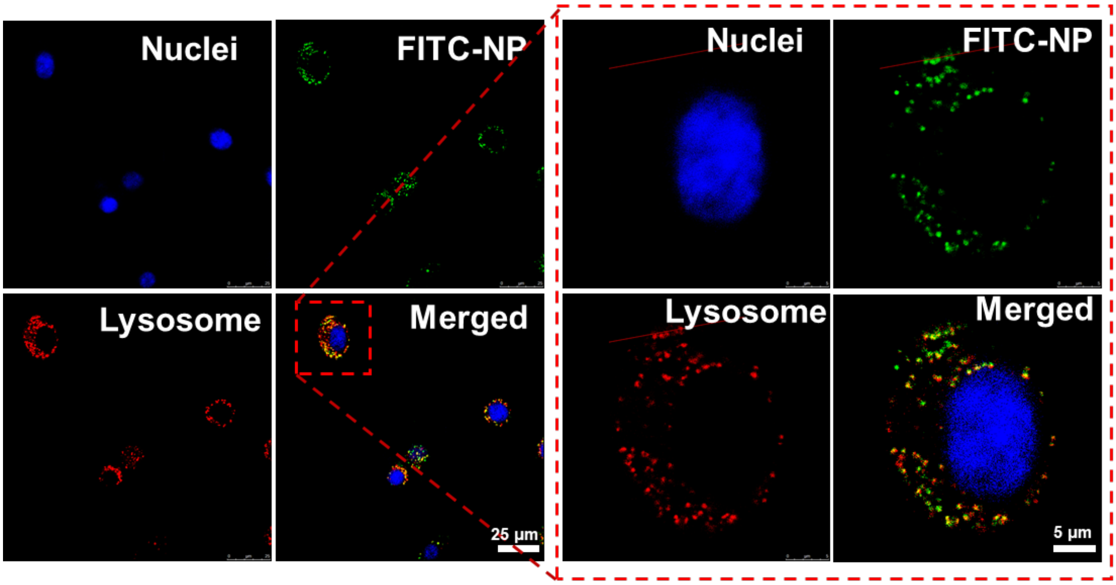

Figure S7. Fluorescence images obtained by CLSM showing cellular uptake of CAMNSN@PSN38 in CT26 cells after incubation for 6 h.

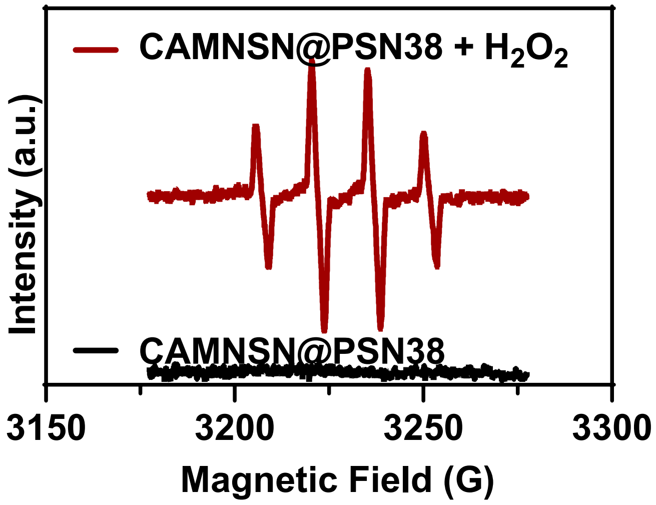

Figure S8. ESR spectra of PBS buffer solution (pH = 5.5) containing CAMNSN@PSN38 with or without the addition of H_2O_2.

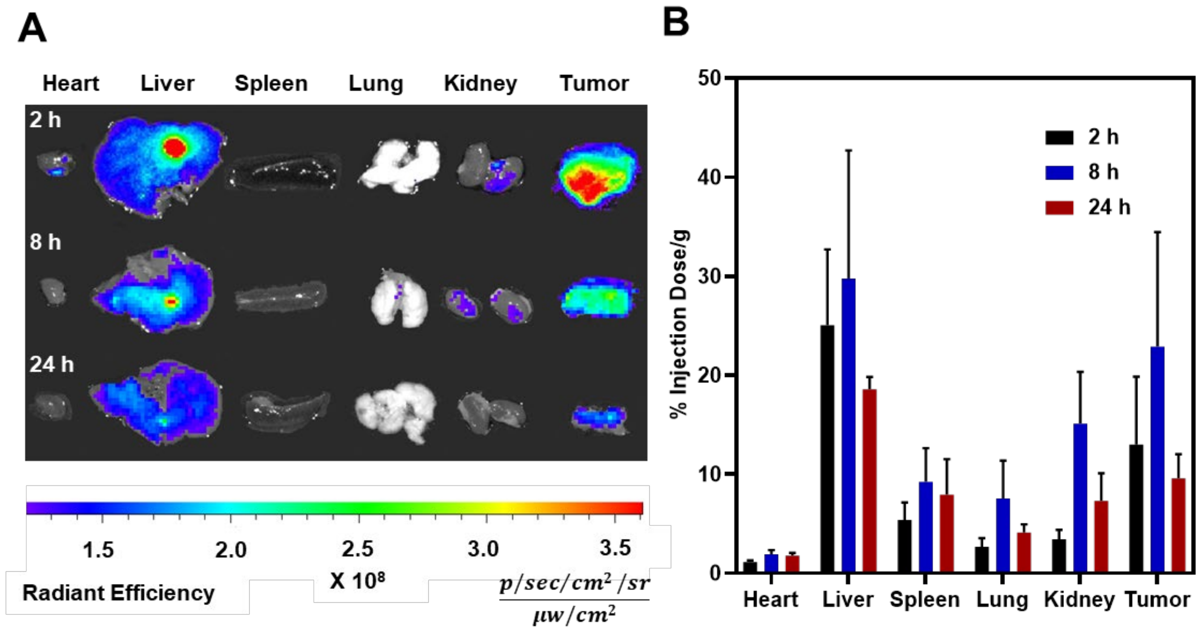

Figure S9. Fluorescence imaging (A) and quantitative biodistribution (B) of major organs and tumor tissues at different time points.

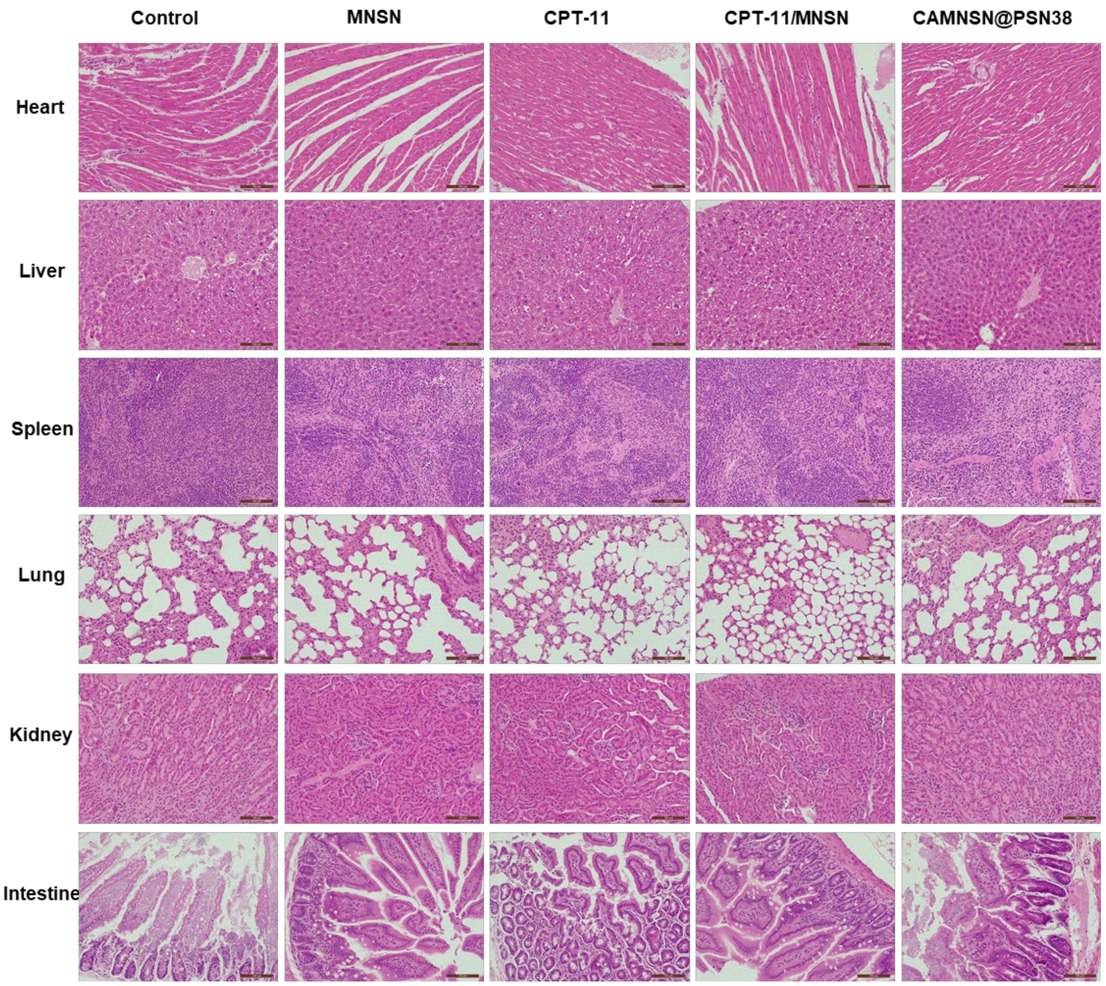

Figure S10. HE staining images of main organs obtained from mice bearing CT26 subcutaneous tumors in each group corresponding to Figure 5C. Scale bar: 100 μm.

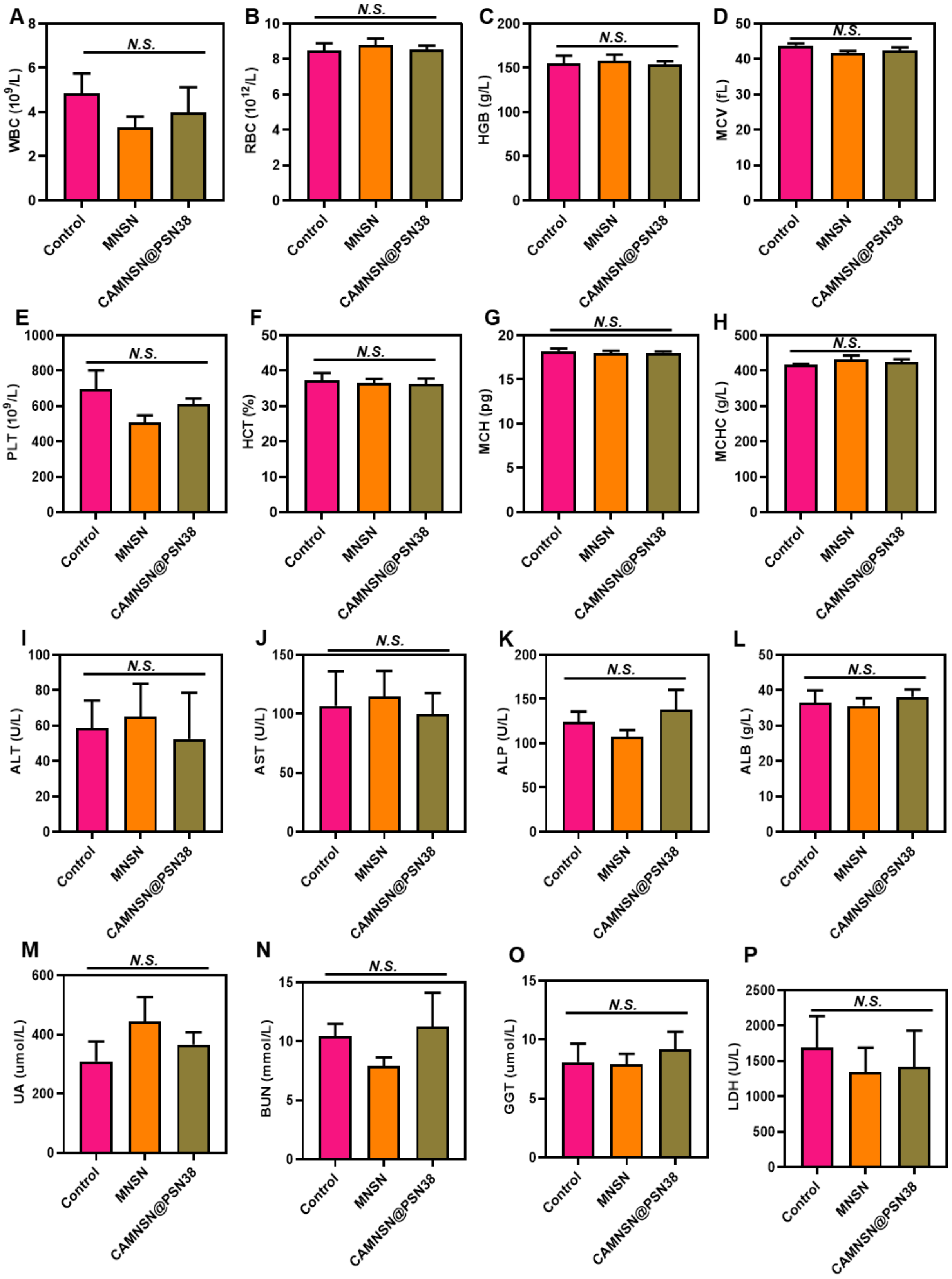

Figure S11. The biosafety evaluation of CAMNSN@PSN38 was tested 24 h after treatments of MNSN (5mg/kg) and CAMNSN@PSN38 (e.q. SN38 concentration: 10 mg/kg, e.q. MNSN concentration: 5mg/kg) treatment. (A) white blood cells (WBC), (B) red blood cells (RBC), (C) hemoglobin (HGB), (D) mean corpuscular volume (MCV), (E) platelet (PLT), (F) hematocrit (HCT), (G)mean corpuscular hemoglobin (MCH), (H) mean corpuscular

hemoglobin (MCHC), (I) alanine aminotransferase (ALT), (J) aspartate aminotransferase (AST), (K) alkaline phosphatase (ALP), (L) albumin (ALB), (M) uric acid (UA), (N) blood urea nitrogen (BUN), (O) γ-glutamyl transpeptidase(GGT) and (P) Lactate dehydrogenase(LDH). (n = 5 biologically independent samples). Data are presented as mean values ± SD.

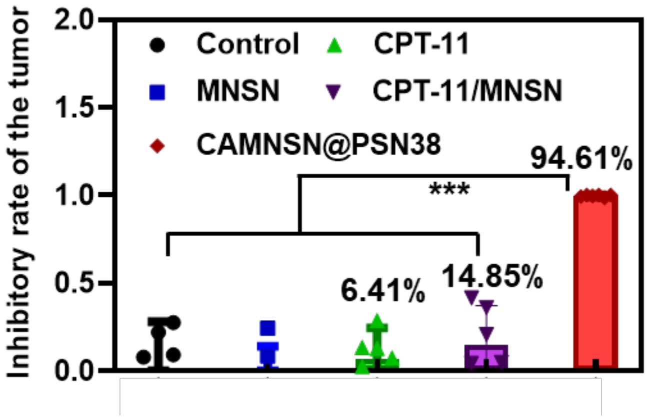

Figure S12. Corresponding inhibitory rate of the CT26 tumor of each group at the end of the experiment in Figure 6C. CPT-11 (e.q. SN38 concentration: 10 mg/kg), MNSN (5mg/kg), CPT-11/MNSN (e.q. SN38 concentration: 10 mg/kg, e.q. MNSN concentration: 5mg/kg) and CAMNSN@PSN38 (e.q. SN38 concentration: 10 mg/kg, e.q. MNSN concentration: 5mg/kg). Treated with tail vein injection every other day for 3 times. n = 6, mean ± SD, ***$p < 0.001$.

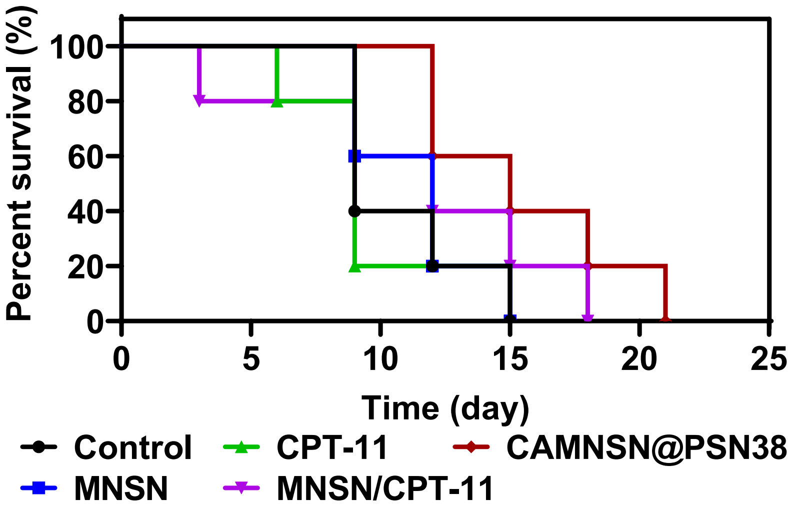

Figure S13. Survival ratio of the mice bearing intraperitoneal CT26 tumor model treated with different treatments. Balb/c nude mice bearing intraperitoneal CT26 tumors were treated with CPT-11 (e.q. SN38 concentration: 10 mg/kg), MNSN (5mg/kg), CPT-11/MNSN (e.q. SN38 concentration:10 mg/kg, e.q. MNSN concentration: 5mg/kg) and CAMNSN@PSN38 (e.q. SN38 concentration: 10 mg/kg, e.q. MNSN concentration : 5mg/kg) with tail vein injection every other days for 3 times. n = 6, mean ± SD.
